# Endothelial cell rearrangements during vascular patterning require PI3-kinase-mediated inhibition of actomyosin contractility

**DOI:** 10.1038/s41467-018-07172-3

**Published:** 2018-11-16

**Authors:** Ana Angulo-Urarte, Pedro Casado, Sandra D. Castillo, Piotr Kobialka, Maria Paraskevi Kotini, Ana M. Figueiredo, Pau Castel, Vinothini Rajeeve, Maria Milà-Guasch, Jaime Millan, Cora Wiesner, Helena Serra, Laia Muixi, Oriol Casanovas, Francesc Viñals, Markus Affolter, Holger Gerhardt, Stephan Huveneers, Heinz-Georg Belting, Pedro R. Cutillas, Mariona Graupera

**Affiliations:** 1 Vascular Signalling Laboratory, ProCURE, Oncobell Program, Institut d´Investigació Biomèdica de Bellvitge (IDIBELL), Gran Via de l’Hospitalet 199, 08908 L´Hospitalet de Llobregat, Barcelona, Spain; 2 Centre for Haemato-Oncology, Barts Cancer Institute, Queen Mary University of London, London, EC1M 6BQ UK; 3 Biozentrum der Universität Basel, Klingelbergstrasse 50/70, 4056 Basel, Switzerland; 4 Helen Diller Family Comprehensive Cancer Center, University of California-San Francisco, 1450 3rd Street, San Francisco, CA 94158 USA; 5 Centro de Biología Molecular Severo Ochoa, CSIC-UAM, Calle Nicolás Cabrera, 28049 Madrid, Spain; 6 Translation Research Laboratory, ProCURE, Oncobell Program, IDIBELL, Gran Via de l’Hospitalet 199, 08908 L´Hospitalet de Llobregat, Barcelona, Spain; 7 Departament de Ciències Fisiològiques II, Universitat de Barcelona, Carrer de la Feixa Llarga, 08907 L´Hospitalet de Llobregat, Barcelona, Spain; 8 Max-Delbrueck Center for Molecular Medicine (MDC), Robert-Rössle-Straße 10, 13125 Berlin, Germany; 9 The German Center for Cardiovascular Research (DZHK), Oudenarder Str. 16, 13347 Berlin, Germany; 10 The Berlin Institute of Health (BIH), Berlin, 10178 Germany; 11 Department of Medical Biochemistry, Amsterdam UMC, University of Amsterdam, Amsterdam Cardiovascular Sciences, Meibergdreef 9, 1105 AZ Amsterdam, Netherlands; 12 CIBERONC, Instituto de Salud Carlos III, Av. de Monforte de Lemos, 5, 28029 Madrid, Spain

## Abstract

Angiogenesis is a dynamic process relying on endothelial cell rearrangements within vascular tubes, yet the underlying mechanisms and functional relevance are poorly understood. Here we show that PI3Kα regulates endothelial cell rearrangements using a combination of a PI3Kα-selective inhibitor and endothelial-specific genetic deletion to abrogate PI3Kα activity during vessel development. Quantitative phosphoproteomics together with detailed cell biology analyses in vivo and in vitro reveal that PI3K signalling prevents NUAK1-dependent phosphorylation of the myosin phosphatase targeting-1 (MYPT1) protein, thereby allowing myosin light chain phosphatase (MLCP) activity and ultimately downregulating actomyosin contractility. Decreased PI3K activity enhances actomyosin contractility and impairs junctional remodelling and stabilization. This leads to overstretched endothelial cells that fail to anastomose properly and form aberrant superimposed layers within the vasculature. Our findings define the PI3K/NUAK1/MYPT1/MLCP axis as a critical pathway to regulate actomyosin contractility in endothelial cells, supporting vascular patterning and expansion through the control of cell rearrangement.

## Abstract (web summary)

Angiogenesis requires dynamic endothelial rearrangements and relative position changes within the vascular tubes. Here the authors show that a PI3K/NUAK1/MYPT1/MLCP pathway regulates actomyosin contractility in endothelial cells and cellular rearrangement during vascular patterning.

## Introduction

Tissue growth and homoeostasis require the establishment of a functional hierarchical tubular network of blood vessels^1^. Blood vessels are mainly formed by a process known as sprouting angiogenesis in which new vascular sprouts arise from parental vessels, grow, and fuse to an adjacent sprout or a pre-existing vessel^1,2^. Newly formed sprouts are highly dynamic with endothelial cells interchanging their relative position within the vascular tube^3–7^. This collective cell migration across the vascular tubes relies on cell rearrangement; yet the regulation of this cell behaviour during the formation and patterning of blood vessels is poorly understood.

Endothelial cell rearrangement occurs through the reorganization of cell–cell junctional contacts thereby allowing the modification of cell−cell adhesion strengths^7–10^. In endothelial cells, there are two types of vascular endothelial-cadherin (Cdh5/VE-cadherin)-based junctional patterns, namely continuous or straight, and discontinuous or serrated^7,9,10^. Straight junctional VE-cadherin organization is mainly found in stable and mature junctions, whereas the serrated VE-cadherin junctional pattern is considered as immature or remodelling junctions^9,10^. Although these VE-cadherin junctional patterns are not visible in the endothelium during zebrafish developmental angiogenesis^11^, computational models have proposed that spatial heterogeneity of these junctional patterns is necessary for cells to rearrange in vivo^7^. Yet, how these junctional profiles impact on the capacity of endothelial cells to rearrange remains poorly understood. Formation, remodelling, and stabilization of cell−cell adhesions in cultured endothelial cells are mediated by actin structures^12^. At mature junctions, linear VE-cadherin is aligned to parallel cortical actin bundles. Instead, serrated immature junctions are connected to perpendicular or radial tensile actin cables^9,10^. The switch between stable and immature junctions is mediated by actomyosin contraction-based pulling forces at the cell–cell junctions^9,10,13^.

Among the different players of the angiogenic process, class I PI3-kinases (PI3K) have emerged as a critical node^14^, for both the physiology of endothelial cells^15,16^ and the pathogenesis of venous malformations^17,18^, the most common type of vascular malformations. PI3Ks are lipid kinases that signal downstream of a variety of cell surface receptors and regulate cellular functions including growth, proliferation, migration, and metabolism^19^. Upon activation, these enzymes generate the lipid phosphatidlylinositiol-3,4,5-triphosphate, a second messenger that triggers signalling pathways, such as those mediated by the serine/threonine kinase AKT and its substrates^20^. Of the class I PI3K isoforms, PI3Kα has been shown to be the only isoform required for endothelial-mediated vascular development^15^. Several studies have demonstrated that PI3Kα signalling primary regulates cell motility during angiogenesis in mouse and zebrafish^15,21,22^.

Our study uncovers a PI3K downstream pathway, namely NUAK1/MYPT1/MLCP, as a critical node in the regulation of cell rearrangement during vessel growth. We have found that blockade of PI3Kα signalling impairs junctional remodelling, inhibits cell rearrangement and drives endothelial cells to grow in superimposed aberrant layers. We identify that a failure of cells to rearrange results in cell stretching and inability to remodel and stabilize new cell–cell contacts upon anastomosis. Through a combination of in vivo and in vitro approaches together with an unbiased and deep quantitative phosphoproteomic screening, we have discovered that PI3Kα mediates cell rearrangement by inhibiting actomyosin contractility through NUAK1/MYPT1/MLCP.

## Results

### PI3Kα mediates rearrangement of endothelial cells

**Fig. 1:**
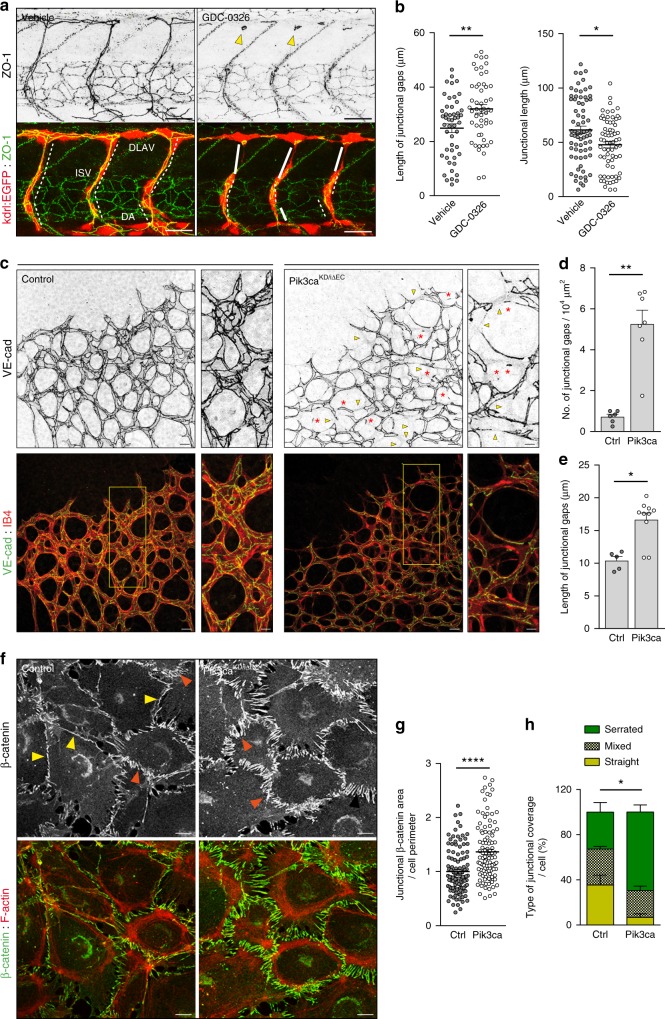
Defects in junctional remodelling upon inactivation of PI3Kα in endothelial cells. **a** Lateral views of intersomitic vessels (ISV) in vehicle and GDC-0326 (50 μM)-treated transgenic Tg(*kdrl:EGFP*)^s843^ (shown in red) embryos stained for ZO-1 (green) at 33 h post fertilization (hpf). Single ZO-1 staining is shown in upper row. DA refers to dorsal aorta and DLAV refers to dorsal longitudinal anastomotic vessels. White lines indicate ZO-1 negative staining; punctuate white lines indicate elongation of junction; yellow arrowheads show ring-shape junctions. **b** Quantification of the length of the dorsal part of the ISVs without ZO-1 (left graph) and length of the ISVs with continuous ZO-1 staining (right graph) in vehicle and GDC-0326 treated embryos (*n* ≥ 54 ISVs per treatment). **c** Representative maximum intensity projections of anti-VE-cadherin (green) and isolectin B4 (IB4, red) immunostained control and *Pik3ca*^*KD/iΔEC*^ mouse retinas at P7. Single channel is shown in upper row. Yellow islets show higher magnification of selected regions shown to the right. Yellow arrowheads indicate vascular segments without VE-cadherin staining; red asterisks indicate VE-cadherin-positive isolated rings or single-dots within the vascular tubes, indicating cell−cell junctional contacts that have not elongated. **d**, **e** Quantitative number of VE-cadherin-negative vessels (junctional gaps) per unit area (*n* ≥ 5 retinas per genotype) (**d**) and length of vessel structures without VE-cadherin (length of junctional gaps) (*n* ≥ 6 retinas per genotype) (**e**). **f** Confocal immunofluorescence images of primary mouse endothelial cells isolated from control and *Pik3ca*^*KD/iΔEC*^ mice stained for β-catenin (green) and F-actin (red) after being treated with 4-OHT for 72 h and re-plated on gelatin-coated slides for 24 h. Yellow arrowheads indicate straight junctional pattern; orange arrowheads indicate serrated junctional pattern. **g** Graph shows the average of β-catenin-positive area along junctional linescans (*n* ≥ 109 cells from six independent experiments). **h** Quantification of percentages of cells with serrated, straight or mixed junctions (*n* = 5 independent experiments). Scale bars, 30 µm (**a**), 20 µm (**c**), 10 µm (**c** small panel, **f**). Data in **b**, **d**, **e**, **g**, **h** represent mean ± SEM (error bars). *P < 0.05, **P < 0.01, ****P < 0.0001 were considered statistically significant. Statistical analysis was performed by the two-sided Mann–Whitney test

Here, we investigated how endothelial cell rearrangements within the vascular sprouts contribute to vessel expansion and patterning. We and others have shown that the PI3Kα isoform regulates endothelial cell motility (Supplementary Fig. 1, Supplementary Movies 1 and 2, and refs. ^15,21,22^). Therefore, we predicted that cell rearrangement would be altered upon blockage of this signalling node. To validate our hypothesis, we first studied vessel growth in zebrafish embryos treated with a PI3Kα isoform-specific inhibitor (GDC-0326; ref. ^23^). We focused our studies between 27 and 38 h post fertilization (hpf), when intersegmental vessels (ISVs) that arise from the dorsal aorta reach the dorsal roof and form the dorsal longitudinal anastomotic vessels (DLAVs)^24^. Treatment with GDC-0326 efficiently inhibited PI3K signalling (Supplementary Fig. 2a) and this led to aberrant endothelial cell junctional patterns. Junctions were frequently disconnected in the dorsal region of the ISV axis and the junctional elongation was reduced in both ISVs and DLAV (Fig. 1a, b). We also observed the presence of ring-shaped junctions in these embryos that suggests lack of tight contact between adjacent endothelial cells (Fig. 1a). Moreover, GDC-0326-treated embryos showed a delay in the growth of ISVs (Supplementary Fig. 2b-d), without an overall delay in embryo development (Supplementary Fig. 2e). These aberrant junctional patterns are indicative of defects in cell rearrangements^11^. Conversely, in vehicle-treated embryos, ISV outgrowth was accompanied by a normal extensive junctional remodelling leading to a pattern of highly elongated endothelial cell junctions (Fig. 1a, b).

In order to translate our observations into a mammalian model, we inactivated PI3Kα in endothelial cells using genetic approaches and we studied retinal angiogenesis. To maximize *Pik3ca* deletion and avoid compensations by other PI3K isoforms we studied *Pik3ca*^*KD/flox*^ mice, in which one *Pik3ca* allele is a constitutive kinase-dead (KD)^25^, and the other is a lox-P-flanked *Pik3ca* allele^15^. *Pik3ca*^*KD/flox*^ mice were crossed into the *Pdgfb-iCreER* transgenic mouse, which expresses a tamoxifen-inducible CRE recombinase specifically in endothelial cells^26^ (further referred to as *Pik3ca*^*KD/iΔEC*^) (Supplementary Table 1 provides details of the mouse models used). To ensure that *Pik3ca flox* allele is completely knocked out in our experimental settings, we assessed PI3Kα half-life in *Pik3ca*^*flox/flox*^ mice crossed into the *Pdgfb-iCreER* (referred to as *Pik3ca*^*iΔEC/iΔEC*^). Complete depletion of the PI3Kα protein was achieved at 96 h after 4-hydroxytamoxifen (4-OHT) treatment (Supplementary Fig. 3a). Thus, to reach complete PI3Kα depletion in vivo, we administered 4-OHT at postnatal day (P) 1 and P2 and investigated *Pik3ca*^*KD/iΔEC*^ retinas at P7. As compared to control retinas, *Pik3ca*^*KD/iΔEC*^ P7 retinas showed a decrease in phospho-S6 (pS6) (S240/4) and an enhanced nuclear FOXO1 staining confirming the inactivation of the PI3K pathway (Supplementary Fig. 3b, c).

In line with our previous findings in zebrafish embryos, we observed that endothelial cells in *Pik3ca*^*KD/iΔEC*^ retinas showed an increased number of junctional gaps (Fig. 1c–e; yellow arrowheads). Furthermore, in a proportion of vascular tubes, the junction gaps resulted either in isolated rings or single-dots of VE-cadherin, indicating lack of tight contacts between endothelial cells (Fig. 1c; red asterisks). To investigate this phenotype in further detail, we analysed VE-cadherin-based cell−cell junctions in cultured endothelial cells from *Pik3ca*^*KD/iΔEC*^ mice by immunostaining for β-catenin and F-actin (Fig. 1f). These experiments showed that *Pik3ca*^*KD/iΔEC*^ endothelial cells failed to establish mature cell−cell junctions; instead, most of the junctions remained immature and connected to radial actin fibres (Fig. 1f–h). Together these data indicate that PI3Kα signalling is involved in cell−cell junctional remodelling in endothelial cells, a process required for cellular rearrangements within the vasculature.

### Defects in cell rearrangements lead to anastomosis failure

**Fig. 2:**
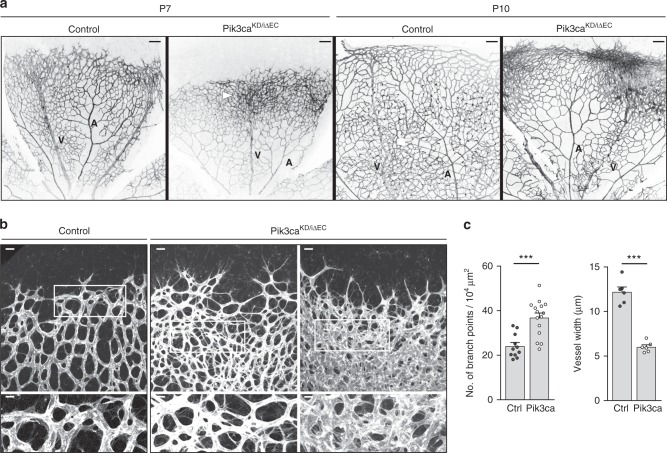
Endothelial PI3Kα regulates vascular growth. **a** Representative images of whole mount retinas stained with IB4 from control and *Pik3ca*^*KD/iΔEC*^ mouse littermates at P7 and at P10. Veins (V) and arteries (A) are indicated. White arrowheads show areas where the vascular plexus is superimposed. **b** Representative high-magnification images of whole mount retinas stained with IB4 from control and *Pik3ca*^*KD/iΔEC*^ mouse littermates at P7. Two independent areas for *Pik3ca*^*KD/iΔEC*^ retinas are shown. White islets show higher magnification of selected regions shown below. **c** Quantification of number of branch points per unit area and vessel width per unit area of control (Ctrl) and *Pik3ca*^*KD/iΔEC*^ (*Pik3ca*) retinas (*n* ≥ 6 retinas per genotype). Scale bars, 100 µm (**a**), 20 µm (**b**, upper panels), 10 µm (**b**, lower panels). Data represent mean ± SEM (error bars). ***P < 0.001. Statistical analysis was performed by the two-sided Mann–Whitney test

P7 *Pik3ca*^*KD/iΔEC*^ retinas exhibited a significant reduction in vascular radial outgrowth (Fig. 2a and Supplementary Fig. 4a) with no detectable differences in sprouting activity (Supplementary Fig. 4c, d). This phenotype was further increased at P10, with the sprouting front of *Pik3ca*^*KD/iΔEC*^ retinas neither reaching the periphery of the retina nor invading deeper retinal layers (Fig. 2a and Supplementary Fig. 4b). However, P7 *Pik3ca*^*KD/iΔEC*^ retinas showed an increase in vessel density with vascular tubes growing in multiple layers (Fig. 2a–c; Supplementary Fig. 4e and Supplementary Movies 3 and 4); this superposition was more pronounced at P10 (Supplementary Fig. 4b, f). Also, the vascular tubes in *Pik3ca*^*KD/iΔEC*^ retinas showed smaller calibre (Fig. 2b, c). Analysis of *Pik3ca*^*KD/WT*^ and *Pik3ca*^*iΔEC/iΔEC*^ retinas (Supplementary Table 1) showed that the former appeared normal, whereas *Pik3ca*^*iΔEC/iΔEC*^ retinas looked similar to *Pik3ca*^*KD/iΔEC*^ retinas but with milder vascular defects (Supplementary Fig. 4a, b).

**Fig. 3:**
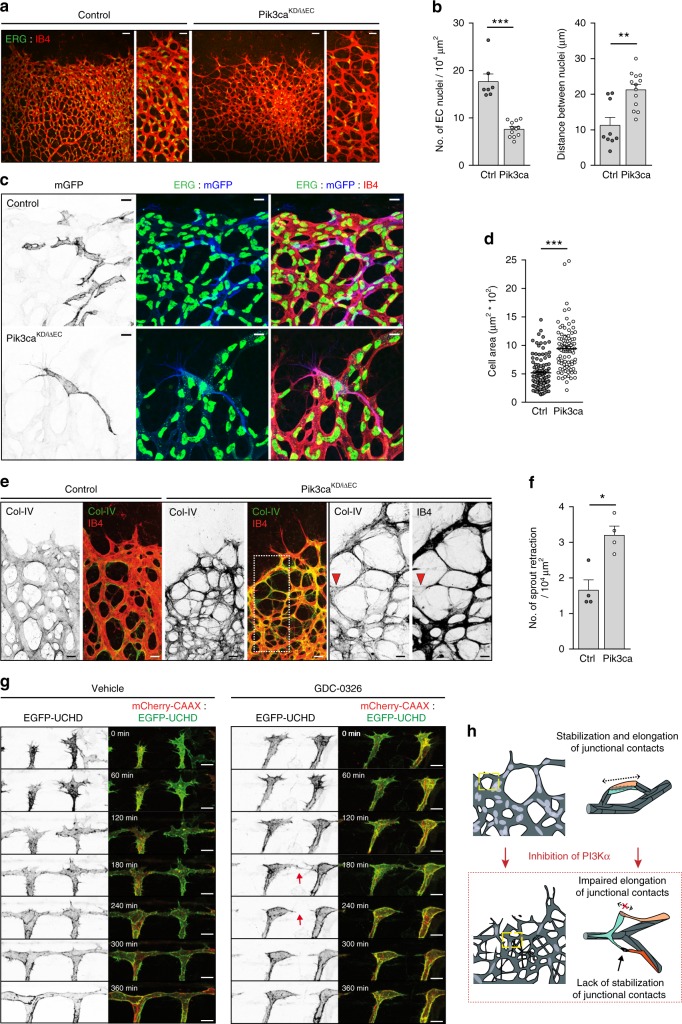
Cell stretching defects and anastomosis failure in *Pik3ca*^*KD/iΔEC*^ vessels. **a** Representative images of retinas stained for ERG (green) and IB4 (red) from control and *Pik3ca*^*KD/iΔEC*^ P7 pups. Higher magnification are shown to the right. **b** Quantification of endothelial cells per unit area assessed by ERG positivity and of the distance between two adjacent endothelial cell nuclei (*n* ≥ 6 retinas per genotype). **c** Immunostaining of single-cell labelling with membrane-bound GFP (mGFP, blue), endothelial nuclei (ERG, green) and blood vessel (IB4, red). Retinas from *Pdgfb-iCre;Pik3ca*^*WT/flox*^ were used as control. **d** Quantification of cell size of individual mGFP-positive cells in control and *Pik3ca*^*KD/iΔEC*^ retinas. At least 89 individual mGFP-positive cells from four independent retinas per genotype were quantified. **e** Images of control and *Pik3ca*^*KD/iΔEC*^ P7 retinas stained for collagen IV (green) and IB4 (red). Single channels are also shown. White punctuated islet in the image of a *Pik3ca*^*KD/iΔEC*^ retina shows higher magnification of selected region to the right. Red arrowheads indicate a retracting sprout. **f** Quantification of retracting sprouts per area (*n* = 4 retinas per genotype). **g** Images from a time-lapse movie (starting at 30 hpf) showing lateral views of ISV morphogenesis in transgenic Tg(*UAS:EGFP-UCHD*)^ubs18^;(*kdrl:mCherry-CAAX)*^S916^ embryos treated with vehicle (left panel) or GDC-0326 (50 μM) (right panel). Endothelial cell membrane is visualized in red and the actin cytoskeleton is visualized by F-actin binding domain of utrophin in green. Single channels are also shown. Red arrow shows a retracting event between two endothelial cells. **h** Schematic illustration showing the vascular defects driven by inactivation of PI3Kα (designed by Ana Angulo-Urarte). During vessel growth remodelling, stabilization, and elongation (punctuated arrow) of adherent junctional contacts is required between neighbouring endothelial cells to rearrange. Upon inactivation of PI3Kα, endothelial cells fail to stabilize (black arrow) and elongate (crossed punctuated arrow) junctional contacts. Scale bars, 40 µm (**a**), 20 µm (**a** amplified panels, **e**), 10 µm (**c**, **e** amplified panels) 15 µm (**g**). Data in **b**, **d**, and **f** represent mean ± SEM (error bars). *P < 0.05, **P < 0.01, ***P < 0.001. Statistical analysis was performed by the two-sided Mann−Whitney test

Current models of enhanced vessel density involve an increase in the number of endothelial cells^16,17,27,28^. However, immunostaining of endothelial cell nuclei revealed a significant reduction in the total number of endothelial cells (Fig. 3a, b) and in endothelial cells in S-phase in *Pik3ca*^*KD/iΔEC*^ retinas (Supplementary Fig. 5a, b). Endothelial cells were highly stretched in *Pik3ca*^*KD/iΔEC*^ retinal vessels, with the distance between adjacent endothelial cell nuclei increased by twofold (Fig. 3a, b). This suggests that these cells failed to elongate alongside each other, a process known as cell pairing^29^, and as a consequence the cells overstretched. Given the lower number of endothelial cells in GDC-0326-treated embryos and in *Pik3ca*^*KD/iΔEC*^ retinas, a reduction in cell number might interfere with cell rearrangement during sprouting angiogenesis (as shown in Fig. 1). To test this possibility, we inhibited cell proliferation with systemic administration of mitomycin C at P5, 48 h before harvesting the retinas at P7, followed by assessing cell proliferation and vascular patterning. As previously described^30^, 5-ethynyl-2′-deoxyuridine (EdU) incorporation showed a prominent block in endothelial cell proliferation in mitomycin C-treated retinas, leading to an overall reduction in endothelial cells (Supplementary Fig. 5c-f). However, no major differences in VE-cadherin junctional pattern were observed (Supplementary Fig. 5e, f). Similar results were observed in zebrafish embryos upon administration of the cell cycle blockers aphidicolin (APH) or hydroxyurea (HU) (Supplementary Fig. 6a-g). These data indicate that inhibition of cell proliferation per se does not impair cell rearrangement.

We hypothesized that the increase in vascular branches observed in *Pik3ca*^*KD/iΔEC*^ retinas (Fig. 2b, c) was the result of stretched endothelial cells extending multiple protrusions. To visualize single-cell morphology, we used R26-mTmG reporter mice, which express a membrane-bound green fluorescent protein (GFP) upon *Cre* recombination^31^. We crossed R26-mTmG reporter mice with *Pik3ca*^*KD/iΔEC*^ mice and reduced the tamoxifen dose in order to induce mosaic inactivation of PI3Kα (Supplementary Fig. 7a). Analysis of single cells confirmed stretched cell-shapes upon inactivation of PI3Kα (Fig. 3c, d). Cell pairing is an important process for the formation of multicellular vascular tubes; thus the absence of cell pairing might result in defects in lumen formation. Staining for intracellular adhesion molecule 2 (ICAM2), a marker of the apical side of vessels, confirmed frequent lumenal gaps in *Pik3ca*^*KD/iΔEC*^ retinal vessels. The lack of ICAM2 staining in *Pik3ca*^*KD/iΔEC*^ retinas was principally observed in thin tubular structures (Supplementary Fig. 7b, c; yellow asterisks), supporting the notion that the nonluminized tubes are only cell protrusions. Inactivation of PI3Kα also led to an increase in collagen IV empty sleeves in newly formed sprout connections (Fig. 3e, f), showing a lack of stabilization of new contacts and a failure to anastomose. In zebrafish embryos treated with GDC-0326, time-lapse analysis of DLAV formation confirmed that initial contacts between adjacent sprouts occurred but they failed to stabilize, resulting in frequent cell retraction and sprout disconnections (Fig. 3g; Supplementary Movies 5 and 6).

Together, our data show that PI3Kα is necessary for cells to rearrange by regulating remodelling, elongation and stabilization of junctional contacts. Lack of cell rearrangements results in stretched endothelial cells which extend multiple protrusions that fail to anastomose and grow in superimposed aberrant layers (Fig. 3h). While proliferation defects likely contribute to impaired cell rearrangement upon inhibition of PI3K, our data indicate that PI3K signalling primarily regulates junctional remodelling and cell rearrangement.

### PI3Kα inhibition upregulates phospho-MYPT1

**Fig. 4:**
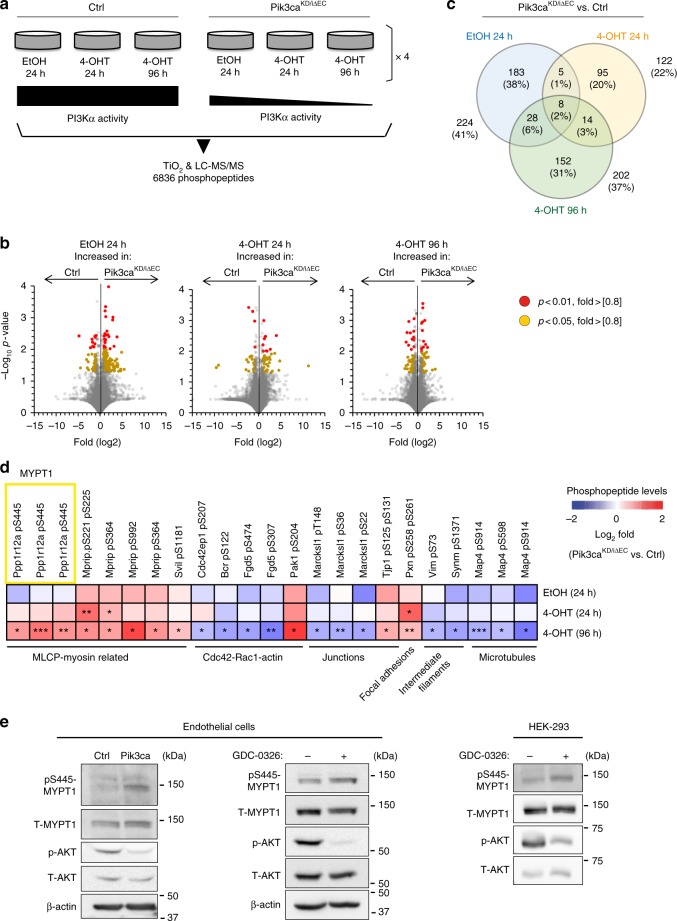
Phosphoproteomics elucidate downstream effectors of PI3Kα in endothelial cells. **a** Schematic illustration of the untargeted label-free mass spectrometry analysis. The study was conducted in *Pik3ca*^*flox/flox*^ (control) and *Pdgfb-iCre;Pik3ca*^*KD/flox*^ *(Pik3ca*^*KD/iΔEC*^) mouse lung endothelial cells under exponential growing conditions upon preincubation with vehicle (EtOH) or 4-OHT for the indicated time points. The vehicle condition for analysis of the heterozygous inactivation of PI3Kα (*Pik3ca*^*KD/flox*^ without induction of CRE activity) was included as a further control and four different mice were analysed in each condition (a total of 24 samples). **b** Volcano plots exhibiting changes in phosphopeptides across genotypes. The *Y* axis represent the negative log_10_ of *P* value and the *X* axis shows the log_2_ of the fold change between control and *Pik3ca*^*KD/iΔEC*^ endothelial cells treated with vehicle (EtOH) for 24 h, 4-OHT for 24 h or 4-OHT for 96 h. Red and yellow dots represent significantly regulated phosphopeptides (*P* < 0.01 and *P* < 0.05 respectively) with a fold-change higher than 0.8 or lower than −0.8. **c** Venn diagram showing the number and percentage of phosphopeptides which are significantly upregulated or downregulated between experimental groups. Number and percentage of overlapping phosphopeptides between groups are also shown. **d** Heatmap indicating fold-changes in the phosphorylation of proteins related to the cytoskeleton. Phosphopeptides identified to be down- or upregulated in *Pik3ca*^*KD/iΔEC*^ vs. Ctrl are shown in blue and red, respectively across EtOH and 4-OHT treatments. Values shown represent mean fold-change over Ctrl. **e** Western blot validation of pS445 MYPT1 in mouse lung endothelial cells and HEK-293 cells upon genetic and pharmacological inhibition of PI3Kα. Control and *Pik3ca*^*KD/iΔEC*^ endothelial cells were treated with 4-OHT for 72 h, re-platted for 24 h and subjected to immunoblotting. Wild-type endothelial cells and HEK-293 cells were treated with vehicle or GDC-0326 for 48 h and subjected to immunoblotting. Quantification of at least three independent experiments is shown in Supplementary Figure 8

To define the molecular and signalling changes occurring upon inactivation of PI3Kα in endothelial cells, we performed untargeted mass spectrometry (MS)-based phosphoproteomics analyses in primary mouse endothelial cells from *Pik3ca*^*flox/flox*^ (control) and *Pik3ca*^*KD/iΔEC*^ mice. We analysed four biological replicates per genotype, each of which was treated with vehicle (ethanol) for 24 h or 4-OHT for 24 and 96 h (Fig. 4a). We identified a total of 6836 phosphopeptides in the 24 samples analysed, which were quantified using a previously described label-free methodology^32^, generating 328,128 data points. This analysis identified: (i) 224 differently regulated phosphopeptides in the *Pik3ca*^*KD/flox*^ endothelial cells relative to control; (ii) 122 deregulated phosphopeptides in *Pik3ca*^*KD/iΔEC*^ endothelial cells upon 24 h treatment with 4-OHT; and (iii) 202 deregulated phosphopeptides in *Pik3ca*^*KD/iΔEC*^ endothelial cells upon 96 h treatment with 4-OHT (Fig. 4b, c).

We clustered the phosphorylation sites that were significantly deregulated at 96 h post 4-OHT treatment by gene ontology (Supplementary Fig. 3a). Of particular relevance for the cell rearrangement phenotype observed, we found a total of 23 phosphosites from 14 cytoskeleton-related proteins that were differently modulated between *Pik3ca*^*KD/iΔEC*^ and control endothelial cells. These included the myosin light chain phosphatase (MLCP), CDC42, RAC1, junctional proteins, focal adhesions, intermediate filaments, and microtubules (Fig. 4d).

Among the different candidates, *Pik3ca*^*KD/iΔEC*^ endothelial cells showed an upregulation on the S445 phosphosite (pS445) of the phosphatase 1 regulatory subunit 12 A (Ppp1r12A) also known as MYPT1 (Fig. 4d). This protein was of particular interest because, together with PP1β and M20, it composes the MLCP complex and regulates the dephosphorylation of the myosin light-chain (MLC) 2 (refs. ^33–36^), which is required for the contraction of actomyosin (illustration is shown in Supplementary Fig. 8a). We validated the increase of pS445 MYPT1 in *Pik3ca*^*KD/iΔEC*^ endothelial cells by western blot (Fig. 4e; Supplementary Fig. 8b, c). Also, we confirmed the PI3Kα-mediated regulation of pS445 MYPT1 in wild-type endothelial and HEK-293 cells treated with GDC-0326 (Fig. 4e; Supplementary Fig. 8b, c).

### PI3Kα is required to suppress actomyosin contractility

**Fig. 5:**
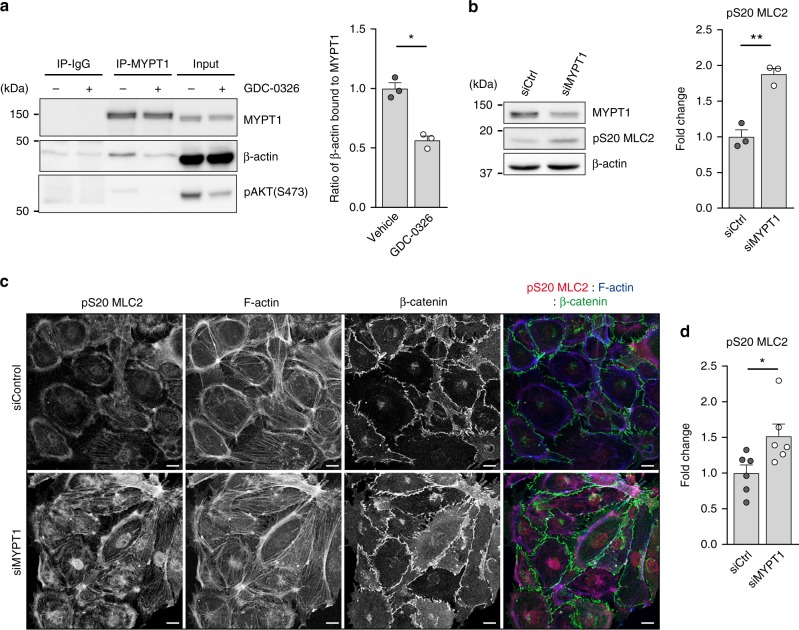
MYPT1 promotes dephosphorylation of MLC2 in endothelial cells. **a** Immunoblot analysis of HEK-293 cells treated with vehicle or GDC-0326 for 48 h using the indicated antibodies. Endogenous MYPT1 was immunoprecipitated and its ability to interact with actin was assessed in an overlay assay. Bars to the right show quantification of actin bound to total MYPT1 from three independent experiments. **b** Western blot analysis of MYPT1, pS20 MLC2 and β-actin in lysates of wild-type mouse lung endothelial cells transfected with siControl (siCtrl) or siMYPT1. Bars to the right show quantification of pS20 MLC2 normalized to β-actin from three independent experiments. **c** Images of endothelial cells transfected with siCtrl or siMYPT1, seeded on gelatin-coated plates 72 h post-transfection, and immunostained for β-catenin (green), pS20 MLC2 (red) and F-actin (blue). **d** Quantification of total cell pS20 MLC2 immunostaining intensity (shown as integrated density) of images shown in **c** (*n* ≥ 6 independent experiments). Scale bars, 15 µm (**c**). Data in **a**, **b**, and **d** represent mean ± SEM (error bars). *P < 0.05, **P < 0.01. Statistical analysis was performed in **a**, and **b** by the two-sided Student’s *t* test and in **d** by the two-sided Mann–Whitney test

Zagoroska et al. found that S445 MYPT1 phosphorylation triggers the binding of MYPT1 to 14-3-3, thereby blocking the ability of the MLCP complex to dephosphorylate MLC2 in S19 and T18/S19 (ref. ^35^). Thus, we predicted that inhibition of PI3Kα, and the resultant increase of MYPT1 S445 phosphorylation, might promote the detachment of MLCP from the actomyosin machinery, thereby increasing actomyosin contractility. Indeed, we detected that the ability of MYPT1 to bind β-actin in an overlay assay was reduced upon inhibition of PI3Kα (Fig. 5a). By knocking down MYPT1 protein expression with small interference RNA (siRNA), we found that phosphorylation of MLC2 at S20 (S19 in humans) was increased upon MYPT1 downregulation in endothelial cells (Fig. 5b–d).

**Fig. 6:**
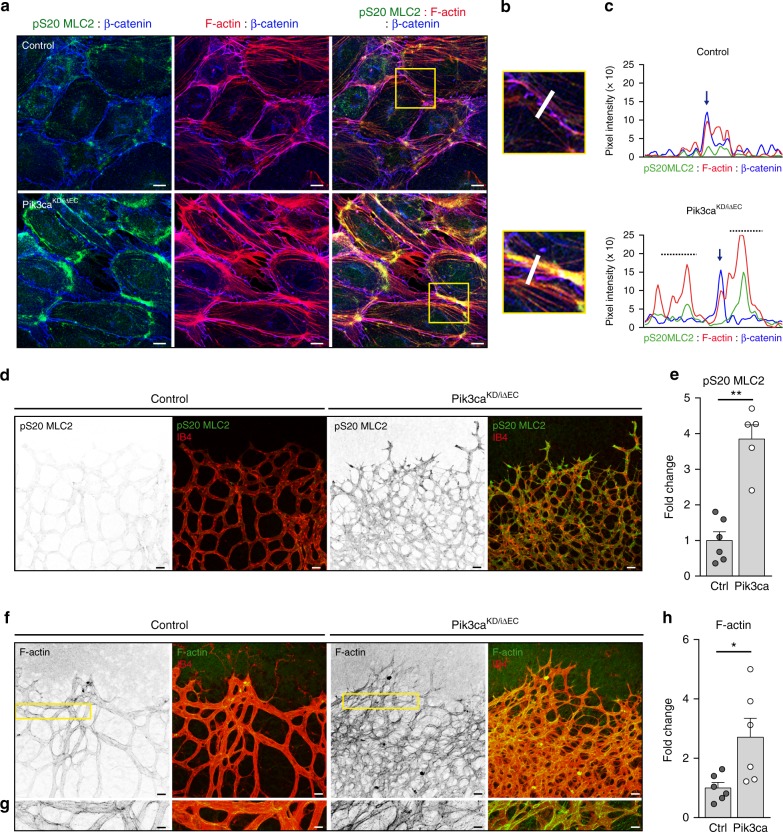
Inactivation of PI3Kα results in increased actomyosin contractility. **a** pS20 MLC2 (green), F-actin (red) and β-catenin (blue) immunostaining of endothelial cells isolated from control and *Pik3ca*^*KD/iΔEC*^ mice treated with 4-OHT for 72 h and re-plated on gelatin-coated slides for 24 h. **b** Yellow islets show higher magnification of selected regions in **a**. **c** Representative fluorescence intensities of pS20 MLC2, β-catenin and F-actin immunostaining corresponding to the area depicted by the white line in **b**. Vertical blue arrows indicate endothelial junction between two endothelial cells. Punctuated lines highlight subcortical area. **d** pS20 MLC2 (green) and IB4 (red) staining of control and *Pik3ca*^*KD/iΔEC*^ P7 retinas. **e** Quantification of the intensity of pS20 MLC2 staining per vascular area (shown as integrated density) (*n* ≥ 5 retinas per genotype). **f** F-actin (green) and IB4 (red) staining of control and *Pik3ca*^*KD/iΔEC*^ P7 retinas. **g** High magnification of selected regions shown in (**f**) illustrates the increase in F-actin intensity induced in *Pik3ca*^*KD/iΔEC*^ endothelium. **h** Quantification of F-actin staining per vascular area (shown as integrated density) (*n* ≥ 6 retinas per genotype). Scale bars, 15 µm (**a**), 20 µm (**d**, **f**), 10 µm (**g**). Data in **e** and **g** represent mean ± SEM (error bars). *P < 0.05, **P < 0.01. Statistical analysis was performed by the two-sided Mann–Whitney test

Enhanced phosphorylation of MLC2 on S20 was also found upon both genetic and pharmacologic inhibition of PI3Kα in mouse endothelial cells (Supplementary Fig. 9a, b). Moreover, pS20 MLC2 staining was enriched at the subcortical region of *Pik3ca*^*KD/iΔEC*^ endothelial cells (Fig. 6a–c; Supplementary Fig. 9c). This was correlated with subcortical accumulation of F-actin (Fig. 6a–c; Supplementary Fig. 9d). Instead, control cells showed stress fibres evenly distributed between the cytoplasm and the subcortical area of the cell (Fig. 6a–c). Analysis of *Pik3ca*^*KD/iΔEC*^ retinal vessels confirmed an increase in the intensity of pS20 MLC2 (Fig. 6d, e) and F-actin staining (Fig. 6f, g). Together, these experiments show that PI3Kα activity is required to downregulate actomyosin contractility and may also explain why *Pik3ca*^*KD/iΔEC*^ endothelial cells fail to stabilize junctions in vivo (Fig. 1).

### NUAK1 inhibition restores PI3Kα-driven vascular phenotypes

**Fig. 7:**
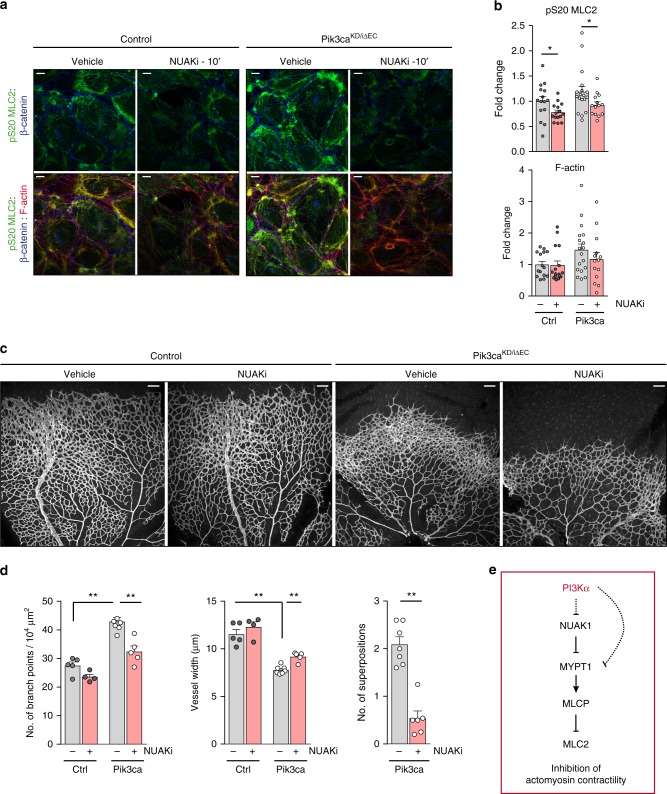
Blockade of NUAK1 restores the endothelial phenotypes imposed by PI3Kα inactivation. **a** Confocal images of control and *Pik3ca*^*KD/iΔEC*^ endothelial cells treated with 4-OHT for 72 h, re-plated on gelatin-coated slides for 24 h and treated with 10 μM WZ4003 (NUAK1 inhibitor; NUAKi) or DMSO as control for 10 min, and stained for pS20 MLC2, β-catenin and F-actin. **b** Quantification of subcortical pS20 MLC2 (upper graph) and F-actin (lower graph) immunostaining intensities (shown as integrated density) (*n* ≥ 14 images of three independent experiments). **c** IB4-stained control and *Pik3ca*^*KD/iΔEC*^ retinas treated with DMSO as control or WZ4003 (NUAKi) at P6 (16:00), and isolated at P7 (1000 hours). **d** Quantification of branch points per unit area, vessel width per unit area, and superimposed vascular tubes per unit area (*n* ≥ 4 retinas per genotype and treatment). **e** Molecular mechanism by which PI3Kα suppresses actomyosin contractility. Scale bars, 15 µm (**a**), 100 µm (**c**). Data in **b** and **d** represent mean ± SEM (error bars). *P < 0.05, **P < 0.01. Statistical analysis was performed by the two-sided Mann–Whitney test

Two non-related protein kinases are able to phosphorylate MYPT1 on S445, namely NUAK family kinase 1 (NUAK1)^35^ and large tumour suppressor kinase 1 (LATS1)^37^. Up to date, only NUAK1 has been reported to regulate phosphorylation of MLC2^35^. Based on these findings, we tested whether restoring MLCP activity by blocking NUAK1 reduces pS20 MLC2 and suppresses actomyosin contractility. Treatment of endothelial cells with a selective NUAK1 inhibitor (NUAKi)^38^ reduced phosphorylation of MLC2 and F-actin staining (Fig. 7a). Inhibition of ROCK, the main kinase that phosphorylates MLC2, was not sufficient to completely abrogate actomyosin contractility in *Pik3ca*^*KD/iΔEC*^ endothelial cells (Supplementary Fig. 10a, b). In contrast, ROCK inhibitor completely abrogated pS20 MLC2 and disrupted stress fibres in control cells (Supplementary Fig. 10a, b). This further supports the role of PI3Kα in the regulation of the phosphorylation of MLC2 in an MYPT1/MLCP-dependent manner.

Next, we attempted to rescue the vascular phenotypes triggered by the inactivation of PI3Kα in vivo by treating newborn mice with NUAKi. While inhibition of NUAK1 did not impact on the outgrowth of the vasculature, it normalized the hyperbranched vascular plexus and the vessel width and prevented the three-dimensional growth in *Pik3ca*^*KD/iΔEC*^ retinas (Fig. 7c, d and Supplementary Fig. 10c). In line with the observations in cultured endothelial cells, administration of ROCK inhibitor in vivo did not rescue the *Pik3ca*^*KD/iΔEC*^ retinal phenotype (Supplementary Fig 10d-f). This further supports that PI3K signalling does not regulate pMLC2 in a ROCK-dependent manner. Altogether, these data demonstrate that regulation of the NUAK1/MYPT1/MLC2 axis by PI3Kα is required to inhibit actomyosin contractility in cultured endothelial cells and in growing vessels in vivo (Fig. 7e).

## Discussion

In this study, we present evidence demonstrating that the PI3K downstream pathway, the NUAK1/MYPT1/MLCP axis, controls actin dynamics in endothelial cells. We uncover that failure of endothelial cells to rearrange results in vascular tubes composed of stretched cells, which grow in a superimposed fashion and fail to stabilize upon anastomosis. Our data support that a tight regulation of endothelial cell movement, beyond tip cells, is necessary for the adequate patterning of the vascular plexus. Cell rearrangement is critical for any process that implies collective cell migration such as in epithelial sheets and neural crest cells during development, and in cancer cells during collective invasion^39,40^. Thus, our discoveries may be translated to the cellular and molecular dynamics orchestrating these critical pathophysiological processes.

The combination of a PI3Kα-selective inhibitor together with an endothelial-specific inducible genetic system has allowed us to fully abrogate PI3Kα activity during vessel development and unravel functions of this signalling hub. We show here that inhibition of PI3Kα hinders endothelial cell pairing and triggers defects in cell elongation. These aberrant cellular behaviours ultimately result in stretches of unicellular vascular tubes devoid of lumen. Thus, our data demonstrate that rearrangement of endothelial cells is required to form well-organized, multicellular vascular tubes. Our experimental strategies also reveal cell proliferation defects in PI3Kα-inhibited endothelial cells. Although it is possible that such defects contribute to the overall phenotype, the observation that blockade of proliferation per se does not interfere with junctional patterns argues for impaired junctional remodelling and cell motility as an independent defect in *Pik3ca*^*KD/iΔEC*^ retinas. Previous data showing that partial decrease in PI3K output selectively decreases endothelial cell migration with no defects in cell proliferation further support this interpretation^15,22^. *Pik3ca*^*KD/iΔEC*^ retinas show an atypical phenotype consisting of hyperbranching but reduced numbers of endothelial cells. In contrast, canonical reduction of endothelial cell proliferation in vivo, by over-activation of Notch or depletion of VEGFR2, results in a hypobranched plexus^41–43^. Our data thus highlight that increased numbers of branches is not always associated with an increase in endothelial cell proliferation as previously described. Taken together, the present data establish that PI3Kα is an important regulator of sprouting angiogenesis and confirm that angiogenic endothelial cells are exquisitely regulated by this signalling hub.

Previous studies have described that PI3K signalling regulates planar cell rearrangement in epithelial cells by stimulating junctional lengthening and stability^44,45^. Our data show that inactivation of PI3K signalling also impairs junctional remodelling in cultured endothelial cells and identify that this also occurs during vessel growth in vivo. Aberrant junctional patterns are indicative of defects in cell rearrangements^11^. Therefore, this suggests that cell rearrangement defects in our PI3Kα-inhibited in vivo models are caused by impaired junctional remodelling. We also identify that the spatial heterogeneity of junctional patterns required for cells to rearrange is lost in *Pik3ca*^*KD/iΔEC*^ endothelial cells, with a concomitant shift towards serrated immature junctions. While current understanding of junctional patterns associates serrated junctions with highly motile cells and straight junctions with non-moving cells^46^, our data suggest that the loss of junctional pattern heterogeneity, regardless of the type, blocks cell rearrangement. This agrees with previous results based on computational models which indicate that cells rearrange when differential adhesion strengths are found between neighbouring cells^7^.

Mechanistically, we show that PI3Kα mediates endothelial cell rearrangement by inhibiting actomyosin contractility at the subcortical edge of the cells. This is in line with previous reports documenting that inactivation of PI3K in vitro leads to both enhanced actin contractility^47^ and aberrant actin pattern^48^. Together, this supports the notion that actomyosin activity has to be low for cell rearrangements to occur. This is not unique of endothelial cells as it has also been observed in *Drosophila* epithelial tracheal tubes and in tumour cells^49,50^. Upon contraction, the actomyosin machinery transmits force to cell−cell contacts and regulates junctional remodelling^12,50^. This process is required for cells to change their adhesion strengths and propel cell movement. We observe that inhibition of PI3K impairs junction stabilization and actomyosin contractility, and in turn reduces cell migration. PI3K is at the crossroad of many extracellular inputs^14^; thus it is not surprising that other mechanisms activate PI3K signalling to stimulate endothelial cell migration during vessel growth^51^. Indeed, during angiogenesis, fibronectin, a critical component of the extracellular matrix of endothelial cells, mediates cell migration through the activation of PI3K^51^. Given that aberrant adhesion of endothelial cells to the extracellular matrix compromises the formation of stable adherent junctions in vivo^52^, we speculate that the regulation of cell−cell adhesion and cell−matrix adhesion by PI3K synergize to mediate cell rearrangement during vessel growth.

The growth of the junctional ring upon establishing new contacts is also impaired upon inactivation of PI3K in both mouse retinas and in zebrafish embryos. This fits with the observation that junctional elongation requires cortical tension from the actomyosin cytoskeleton^11,53^. We postulate that during angiogenesis, a tight equilibrium between stimulation and suppression of actomyosin contractility is required to allow proper growth of the newly formed junctional contact points. This also explains why reduced phosphorylation of MLC2 is associated with junctional defects in angiogenesis^54,55^. Together, our data indicate that the regulation of junctional remodelling and stability by PI3Kα signalling underscores changes in both cell−cell adhesion properties and junctional growth upon new cell−cell contacts. Given the similarity between the vascular defects in *Pik3ca*^*KD/iΔEC*^ retinas and GDC-0326-treated zebrafish and those observed in *cdh5*-mutant zebrafish embryos and mouse retinas, we propose that PI3Kα and VE-cadherin cooperate to regulate cell rearrangement and contact expansion.

We have identified a molecular pathway regulated by PI3K signalling that points towards a role of PI3K in regulating actin dynamics. Specifically, we have discovered that inactivation of PI3Kα leads to enhanced phosphorylation of MYPT1 at S445, a site which can be phosphorylated by NUAK1 (ref. ^35^). We demonstrate that blocking NUAK1 restores both actomyosin contractility and the vascular patterning defects triggered by inactivation of PI3Kα In contrast, NUAKi does not restore the outgrowth of the vasculature in *Pik3ca*^*KD/iΔEC*^ retinas. This might be explained by the inability of NUAKi to restore endothelial cell proliferation; thereby further supporting that cell rearrangement defects and altered cell proliferation in *Pik3ca*^*KD/iΔEC*^ retinas are two non-related events. At the moment, it is still not fully clear how PI3K signalling inhibits NUAK1. Given that AKT inhibits liver kinase B1 (LKB1) through phosphorylation-dependent nuclear retention^56^ and LKB1 activates NUAK1, it is tempting to speculate that PI3Kα blocks the ability of LKB1 to phosphorylate NUAK1. By preventing the phosphorylation of MYPT1 on S445 by NUAK1, PI3Kα then promotes MLCP phosphatase activity (Fig. 7e). Supporting this idea, genetic endothelial depletion of LKB1 opposes *Pik3ca*^*KD/iΔEC*^ vascular phenotypes, with enhanced retinal angiogenesis and increased endothelial cell proliferation and migration^57^. However, taking into account the complexity of the PI3K signalling cascade, other pathways may also contribute to the vascular defects seen in the *Pik3ca*^*KD/iΔEC*^ vasculature.

Taken together, our study discovers the PI3Kα/MYPT1/MLCP signalling axis as a critical hub in endothelial cell rearrangement and highlights the key role of cell rearrangement in the orchestration of collective cell migration during angiogenesis. The regulation of actomyosin remodelling by PI3K signalling has been observed in a variety of primary and tumour cells; yet the molecular mechanism behind this regulation has not been fully understood. Therefore, our findings may be translated into other developmental and pathological situations.

## Methods

### Reagents

All chemicals, unless otherwise stated, were from Sigma-Aldrich. Growing mediums for cultured cells were from Gibco.

### Zebrafish

Maintenance of zebrafish (*Danio rerio*) and experimental procedures involving zebrafish embryos were carried out at the Biozentrum/Universität Basel according to Swiss national guidelines of animal experimentation (TSchV). Zebrafish lines were maintained under licences 1014 H and 1014G1 issued by the Veterinäramt-Basel-Stadt. The fish were maintained using standard procedures and embryos obtained via natural spawning^58^, and embryos were staged by hours post-fertilization (hpf) at 28.5 (ref. ^59^). All experiments were performed in accordance with federal guidelines and were approved by the Kantonales Veterinäramt of Kanton Basel-Stadt (Switzerland). The following zebrafish lines were used: Tg(*kdrl:EGFP-nls*)^ubs1^ (ref. ^29^), Tg(*fli1ep:mCherry-nls*)^ubs10^ (ref. ^60^), Tg(*kdrl:EGFP*)^s843^ (ref. ^61^), Tg(*kdrl:mCherry-CAAX*)^s916^ (ref. ^62^), and Tg(*UAS:EGFP-UCHD*)^ubs18^ (ref. ^11^). For pharmacological inhibition of PI3Kα, dechorionated zebrafish embryos were treated with dimethylsulphoxide (DMSO) or 50 μM GDC-0326 inhibitor (Genentech^23^) in E3 medium supplemented with 0.003% 1-phenyl-2-thiourea from 22 hpf or 27 hpf, as indicated in the Results section. E3 medium containing DMSO or 50 μM GDC-0326 was changed every 4 h. To overcome unspecified off-target effects induced by the combination of PI3Kα inhibitor and laser exposure, all experiments were done in a p53 morpholino background. P53 morpholino (Gene Tools) was dissolved in DEPC water containing 0.2% phenol red and 5 ng were injected to 1−2 cell-stage embryos. In order to block cell cycle during angiogenesis^63^, 26−28 hpf zebrafish embryos were treated with 20 mM hydroxyurea (HU) and 150 μM aphidicolin (APH). Embryo treatment lasted throughout live imaging or until the embryos were fixed in 2 % PFA and prepared for immunofluorescence.

### Mice

Mice were maintained under specific pathogen-free conditions and kept in individually ventilated cages. Experiments were conducted in accordance with the guidelines and laws of the Catalan Departament d’Agricultura, Ramaderia i Pesca (Catalunya, Spain) under the Project License number: DMAH 6809, following protocols approved by the local Ethics Committees of IDIBELL-CEEA. All PI3Kα mutant mice and littermate controls were bred in the C57/BL/6 genetic background (Supplementary Table 1). For the analysis of angiogenesis in the postnatal mouse retina, CRE-mediated recombination was induced in newborn mice by intraperitoneal (i.p.) injections of 25 µg of 4-OHT (2.5 µl of a 10 mg ml^−1^ solution in absolute ethanol) at P1 and P2. Eyes were harvested at P7 and P10 for analysis. Control animals were littermate *Pik3ca*^*flox/flox*^ pups without CRE expression and injected with 4-OHT. For mosaic inactivation of PI3Kα, R26-mTmG reporter mouse^31^ was crossed with *Pik3ca*^*KD/iΔEC*^ mice and 0.8 µg of 4-OHT (2.5 µl of a 0.33 mg ml^−1^ solution in absolute ethanol) was injected i.p. at P1, and eyes were harvested at P7. CRE-mediated recombination was assessed by the expression of membrane-bound GFP. Injected retinas from R26-mTmG;*Pdgfb-iCreER*;*Pik3ca*^*flox/WT*^ mice were used as control for the analysis. The R26-mTmG allele was kept heterozygous. To block proliferation, wild-type pups were i.p. injected with 10 μg g^−1^ of animal of mitomycin C solution (Sigma, #M4287) as described in ref. ^30^ at P5. At P7, all pups were i.p. injected with EdU (Invitrogen) to assess proliferation (EdU staining protocol below). For pharmacological rescue experiment studies the following protocols were used: ROCK was inhibited in half of the pups from the same litter by i.p. injection of 30 mg kg^−1^ of animal of Y-27632 (Calbiochem, #688000) dissolved in DMSO at P6 (18:00) and P7 (10:00), and eyes were harvested at P7 (14:00). NUAK1 was inhibited in half of the pups by a single subcutaneous injection of 30 mg kg^−1^ of animal of WZ4003 (Selleckchem, #S7317) at P6 (16:00 hours), and eyes were harvested at P7 (10:00). Control mice were injected with DMSO only.

### Cells

Mouse lungs were digested with Dispase (Life Technologies, #17105-041; 4 units ml^−1^) for 1 h at 37 °C, followed by positive selection with anti-mouse vascular endothelial-cadherin (Pharmingen, #555289) antibody coated with magnetic beads (Dynal Biotech, #110-35). Cells were seeded on a 12-well plate and were coated with gelatin (0.5%) in DMEM/F12 supplemented with 20% foetal calf serum and EC growth factor (PromoCell, #C30140) and 1% penicillin/streptomycin. After the first passage, the cells were re-purified with vascular endothelial-cadherin antibody-coated magnetic beads. Cells were cultured until passage 5. Human umbilical vein endothelial cells (HUVECs, Lonza, #C2519A) were cultured in EBM-2 culture medium supplemented with EGM-2 BulletKit (Lonza, #CC-3162) on 0.5% gelatin-coated plates and culture up to passage 5. Human embryonic kidney cells (HEK-293, ATCC, CRL-1573) were cultured in DMEM (Lonza, #12-733F) supplemented with 10% of inactivated FBS and 1% penicillin/streptomycin. To induce gene deletion in mouse lung endothelial cells 4-OHT (1 µM) or vehicle (ethanol) was added to the cultured medium for 24 h, followed by replacing the medium without 4-OHT or vehicle. All the experiments were performed 96 h after the addition of 4-OHT or vehicle. For pharmacological inhibition of ROCK and NUAK1 kinases, cells were cultured for 24 h followed by treatment with vehicle (DMSO), 10 μM ROCK inhibitor for 10 or 30 min, or 10 μM NUAK1 inhibitor for 10 min. For pharmacological inhibition of PI3Kα, cells were treated with vehicle (DMSO) and 1 μM GDC-0326 inhibitor (Genentech) for 48 h.

### siRNA transfection

Solution A (493 μl of Opti-MEM (Gibco, #51985026) with 7.5 μl of 20 μM control (Dharmacon, #D-001206-13) or 20 μM MYPT1 (Dharmacon, #M-063177-02 SMART pool siRNA oligomer)) and solution B (493 μl of Opti-MEM with 7.5 μl of lipofectamine RNAi Max (Thermo Fisher Scientific, #13778075)) were prepared and incubated for 5 min at room temperature (RT) in separated tubes following the manufacturer’s instructions. Subsequently, solution B was added to solution A and incubated for 20 min at RT, followed by adding A + B solution to endothelial cells (750,000 cells) in suspension resuspended in 500 μl of Opti-MEM without antibiotics on cover-slips in six-well plates (three cover-slips per well previously coated with 0.5% gelatin). The medium was changed the day after, and cells were either fixed for immunofluorescence assays or lysed for western blotting 72 h after transfection.

### Protein extraction, immunoprecipitation and immunoblotting

Zebrafish embryos were subjected to a deyolking protocol to avoid the interference of yolk proteins in our analyses. Dechorionated embryos were collected in a 1.5 ml tube filled with 1 ml deyolking buffer (55 mM NaCl, 1.8 mM KCl and 1.25 mM NaHCO_3_). By pipetting with a narrow tip the yolk sac was disrupted. The embryos were shaken for 5 min at 200 × *g* to dissolve the yolk. Cells were pelleted at 300 × *g* for 30 s and the supernatant discarded. Two additional washes were performed by adding 1 ml of wash buffer (110 mM NaCl, 3.5 mM KCl, 2.7 mM CaCl_2_, 10 mM Tris/Cl pH 8.5), shaking 2 min at 200 × *g* and pelletting the cells as before. For protein extraction, deyolked zebrafish embryos, mouse lung endothelial cells and HEK-293 cells were lysed in 50 mM Tris HCl pH 7.4, 5 mM EDTA, 150 mM NaCl and 1 % Triton X-100 supplemented with 2 mg ml^−1^ aprotinin, 1 mM sodium fluoride, 1 mM pepstatin, 1 ng ml^−1^ leupeptin, 1 mM phenylmethysulfonylfluoridem, 10 g ml^−1^ Na-p-tosyl-l-lysine chloro-methyl ketone hydrochloride, 1 mM sodium orthovanadate, 1 μM okadaic acid and 1 mM DTT followed by clearance of lysates by centrifugation. Supernatants were resolved on 8, 10 or 12% SDS–PAGE gels, transferred onto nitrocellulose or PVDF membranes. Membranes were blocked in 5% (w/v) skimmed milk, incubated with specific primary antibodies overnight at 4 °C in 2% BSA in 0.1% Tween-20 TBS buffer (further referred to as TBST), then washed three times with TBST and incubated with peroxidase-conjugated secondary antibodies in 5% (w/v) skimmed milk in TBST at RT for 1 h. The following primary antibodies were used: p-AKT (Ser 473) (Cell Signaling Technology, #4060, diluted 1:1000), AKT (Cell Signaling Technology, #9272, diluted 1:2000), VE-cadherin (Santa Cruz Biotechnology, #sc-6458, diluted 1:500), p85 (Millipore, #ABS234, diluted 1:2000), PI3Kα (monoclonal clone U3A^64^), Ser445 MYPT1 (MRC Reagents, #S508C^35^, 1 μg ml^−1^), MYPT1 (MRC Reagents, #S110D, ref. ^35^, 1 μg ml^−1^ with a 10 μg ml^−1^ of a dephosphopeptide variant of the antigen used to raise the antibody), pS19/S20 MLC2 (Rockland Antibodies, #039600-401-416, diluted 1:500), β-actin (Abcam, #ab49900, diluted 1:10,000) and α-tubulin (Sigma-Aldrich, #T6074, diluted 1:10,000). The following secondary antibodies from DAKO were used in a 1:5000 dilution: swine anti-rabbit (#P0399), rabbit anti-goat (#P0449), rabbit anti-mouse (#P0260), and rabbit anti-sheep (#P0163).

MYPT1 immunoprecipitation (IP) was performed using a sheep polyclonal MYPT1 antibody (MRC Reagents, #S110D) covalently coupled to protein G-Sepharose (GE Healthcare, #17-0618-01) (1 μg of antibody per 1 μl of beads) with a dimethyl pimelimidate cross-linking procedure^35^. Cells treated with DMSO or GDC-0326 for 48 h were lysed with IP buffer (50 mM Tris pH 8.0, 150 mM NaCl, 0.1% SDS, 1% NP40, 0.5% sodium deoxycolate, 2 mg ml^−1^ aprotinin, 1 mM pepstatin A, 1 ng ml^−1^ leupeptin, 10 g ml^−1^ TLCK, 1 mM PMSF, 1 mM NaF, 1 mM NaVO_3_ and 1 μM okadaic acid) and clarified by centrifugation at maximum speed for 15 min at 4 °C. Cell lysate (1 mg) was incubated with 5 μg of coupled antibody for 1 h at 4 °C. Immunoprecipitates were washed four times with IP buffer and resuspended in 1× SDS Sample Buffer. Immunoprecipitates and cell lysates (50 μg) were subjected to electrophoresis on 8−12% SDS–PAGE and transferred to nitrocellulose membranes. For sheep antibodies, the membranes were incubated for 30 min with TBST containing 10% (w/v) skimmed milk. The membranes were then immunoblotted in 10% (w/v) skimmed milk in TBST with the MYPT1 primary antibody (1 μg ml^−1^) overnight at 4 °C. The incubation with phosphospecific MYPT1 sheep antibody was performed with the addition of 10 μg ml^−1^ of a dephosphopeptide variant of the antigen used to raise the antibody. Uncropped scans are shown in Supplementary Figures 11-13.

### Live 2D wound healing assay

Mouse lung endothelial cells and HUVECs were plated on six-well plate dishes coated with 0.5% gelatin to grow to confluence for 24 h. Cell monolayers were scratched with a p200 pipette tip to induce cell migration. Phase-contrast images were performed every 10 min using a widefield microscope (NIKON Eclipse TI) equipped with a ×10air objective and an Andor Zyla 4.2 plus sCMOS camera. An Okolab cage incubator and humidified CO_2_ gas chamber set to 37 °C and 5% CO_2_ were used during the imaging process.

### Immunofluorescence analysis in zebrafish, retinas and cells

For immunofluorescence and imaging of zebrafish embryos, dechorionated zebrafish embryos were fixed in 2% paraformaldehyde and 0.1% Tween 20 in PBS overnight at 4 °C. After fixation, embryos were washed four times with 0.1% Tween 20 in PBS (hereafter refer to as PBST) for 5 min, permeabilized with 0.5% Triton-X-100 in PBS at room temperature (RT) for 15−30 min, and blocked with 1% BSA, 5% goat serum (Gibco, #16210-064) and 0.2% Triton X-100 in PBS at 4 °C overnight by continuous shaking. Thereafter, embryos were incubated with mouse anti-human ZO-1 (Zymed, #33-9111, diluted 1:200) in 500 μl of blocking buffer at 4 °C overnight, washed in PBST at least six times for over 3 h, and incubated with Alexa-633 goat anti-mouse IgG (Invitrogen, #A-21053, diluted 1:1000) in 500 μl of blocking buffer overnight at 4 °C. Embryos were finally washed in PBST at 4 °C overnight with continuous shaking. Fixed or live fluorescence positive zebrafish embryos were selected using a Leica MZ FLIII fluorescent, anaesthetized in E3 supplemented with 1× tricaine pH 7 (0.08%) and mounted in a 35 mm glass-bottomed dish (0.17 mm; MatTek), using 0.7% low melting agarose containing 1× tricaine. For live imaging, the mounting agarose was additionally supplemented with 0.003 % phenylthiourea (PTU) and inhibitor if the case.

Mouse eyes were fixed in 4% PFA in PBS for 1 h on ice and washed in PBS for at least 10 min. Retinas were isolated and fixed with 4% PFA in PBS for 1 h on ice. After washing the retinas with PBS three times, they were incubated with blocking buffer (1% BSA, 0.3% Triton X-100 in PBS) overnight at 4 °C. Then, retinas were incubated with the specific primary antibodies diluted in blocking buffer overnight at 4 °C. Primary antibodies against the following proteins were used: pSer240/244-S6 (Cell Signaling Technology, #2215, diluted 1:100), FOXO1 (Cell Signaling Technology, #2880S, diluted 1:100), VE-cadherin (BD Bioscience, #555289, diluted 1:50), ERG (Abcam, #AB92513, diluted 1:400), Collagen IV (Chemicon international, #AB756P, diluted 1:50), ICAM-2 (BD Bioscience, #553326, diluted 1:100), pS19/S20 MLC2 (Rockland Antibodies, #039600-401-416, diluted 1:100). Retinas were washed three times in PBST and then in Pblec buffer (1% Triton X-100, 1 mM CaCl_2_, 1 mM MgCl_2_ and 1 mM MnCl_2_ in PBS, pH 6.8) for 30 min at RT. Thereafter the retinas were incubated for 2 h at RT or overnight at 4 °C in Pblec buffer containing Alexa-conjugated secondary antibodies (diluted 1:200) and Alexa-conjugated Isolectin GS-B4 (IB4, Invitrogen, #I21411, #I21412, I32450, diluted 1:300), washed three times with PBST and flat-mounted on microscope glass slides with Mowiol (Calbiochem, #475904). For F-actin staining, Alexa-Fluor 568-conjugated phalloidin (Invitrogen, #A12380, diluted 1:400) was used. The following secondary antibodies Alexa-Fluor conjugated were used (1:300 dilution): Alexa-Fluor 488 goat anti-rat (Invitrogen, #A11006), Alexa-Fluor 488 goat anti-rabbit (Invitrogen, #A11008), Alexa-Fluor 568 goat anti-rabbit (Invitrogen, #A11011), Alexa-Fluor 647 donkey anti-rabbit (Invitrogen, #A31573). The labelling of proliferative endothelial cells with EdU was performed at P7. Pups were injected i.p. with 60 μl of EdU (0.5 mg ml^−1^ in PBS, Invitrogen, #C10340) 2 h before they were sacrificed. EdU-positive cells were detected in the retinal vasculature with the Click-iT EdU Alexa Fluor-647 Imaging Kit (Invitrogen, #C10340).

Endothelial cells were seeded on cover-slips in six-well plates coated with 0.5% gelatin. 24 h later, corresponding treatment was done and cells were washed once with cold PBS (supplemented with 1 mM CaCl_2_ and 0.5 mM MgCl_2_) and fixed in 4% PFA for 15 min at RT. Cells were then washed with PBS and permeabilized in 0.1% Triton X-100 in PBS for 30 min at RT and incubated in blocking solution (3% BSA, 5% goat serum, 0.1% Triton X-100 in PBS) for 1 h at RT. Cover-slips were removed from the culture plate and were incubated in the appropriate dilution of primary antibodies in blocking solution ON at 4 °C in a wet chamber. Primary antibodies against the following proteins were used: pS19/S20 MLC2 (Rockland Antibodies, #039600-401-416, diluted 1:100) and β-catenin (BD Bioscience #610153, diluted 1:200). The following day, cover-slips were washed three times with PBS at RT and incubated in the appropriate dilution of Alexa-Fluor 488 goat anti-rabbit (Invitrogen, #A11008, diluted 1:200), Alexa-Fluor 568 goat anti-mouse (Invitrogen, #A21236, diluted 1:200) and Alexa-Fluor 633-conjugated phalloidin (Invitrogen, #A22287, diluted 1:400) in PBS for 2 h at RT in a wet chamber. Three washes with PBS were performed, adding 1 μg ml^–1^ of 4′,6-diamidino-2-phenylindole (DAPI; Molecular Probes, #D1306) in the last one.

### Imaging analysis and quantification

All quantifications obtained from confocal (Leica SP5) or widefield microscopes (Nikon Eclipse 80i or Nikon Eclipse TI) were performed using Image J software (http://fiji.sc/). Zebrafish movies were analysed with ImageJ software. Photoshop and Illustrator (Adobe) software were used for image processing.

In the zebrafish, images were taken at ×40(NA = 1.1) water immersion objective, maximum intensity projections were used for quantification and five ISVs were quantified per embryo. Measurements of ISVs length were made straight from the edge of the aorta to the leading edge of the sprout and the number of endothelial nuclei in that length was quantified. The number of endothelial nuclei in the DLAV and DA was quantified per segment within five ISVs. Junctional length and junctional gaps length were measured in embryos stained for ZO-1. Only flat-mounted embryos were selected for quantification avoiding crooked ones.

In the retina, vascular parameter were quantified in at least four images of comparable vascular areas per retina and of at least three mice of each genotype or experimental conditions. All images shown in the figures are maximum intensity projections unless otherwise specified. For the quantification of retinal vessel progression overview, widefield images of IB4-stained retinal vasculature were obtained with the ×10 objective (Nikon Eclipse 80i microscope). The distance of vessel growth from the centre of the optic nerve to the edge of the angiogenic front was measured per each retina leaflet. The mean of all leaflet measurements was obtained per retina and compared between control and mutant groups. For all other quantifications high-resolution confocal images at ×40 oil immersion objective was used. The number of filopodia and sprouts were quantified at the angiogenic front. The total number of filopodia and sprouts were normalized to a vessel length of 100 μm at the angiogenic front. Endothelial branch points, vessel width, superimposed vascular tubes, number of junctional gaps, length of junctional gaps, distance between nuclei, endothelial cell numbers, collagen IV empty sleeves and lumen disconnections were quantified behind the angiogenic front in fields sized 100 μm × 100 μm. For quantification of cell shape, single GFP-positive cells located behind the sprouting front were only considered. Vessel width, distance between neighbouring cells, length of junctional gaps and area of GFP-positive cells was determined using ImageJ software with the proper scale set up. To assess the proliferation rate, double-positive EdU/ERG endothelial nuclei were counted in field of 200 μm × 200 μm behind the vascular sprouting front and the total number was divided by the total number of endothelial cells (ERG-positive nuclei). For quantification of pS20 MLC2 and F-actin immunostaining, signal intensity within IB4 positive area in images taken with the ×40 oil immersion objective was measured. In brief, manual threshold was set to obtain binary images of IB4 staining. IB4-positive area was measured and defined as a region of interest (ROI). Integrated density of pS20 MLC2 or F-actin was measured in the IB4-positive area for each image. Then, to calculate the corrected total fluorescence (CTF), the following formula was used: CTF = Integrated Density – (Area selected for IB4 positivity × Mean fluorescence of background readings). The background readings were taken from three areas close to the vasculature but negative for IB4. 3D reconstructions were generated by using the Leica LAS-X.

In phase-contrast wound healing assays, quantification of cell migration was made by measuring the percentage of cell-free area. Cells from the first, second and third row were manually tracked using the Manual Tracking plugin and cell velocity, directionality and travelled distance were calculated with the chemotaxis tool plugin.

In cultured endothelial cells, maximum intensity projections of confocal images with the ×63 oil immersion objective were acquired. Signal intensity of pS20 MLC2 and F-actin immunostaining/cell was quantified using the junctional staining of β-catenin to select individual endothelial cells (ROI A1). Using the enlarge command two concentric areas were drawn in each cell (distance of −2.5 μm (ROI A2) and −7.5 μm (ROI A3) from the junctional β-catenin positive staining). Integrated density was measured. For measuring total intensity levels: area from ROIs A1 was used as templates to measure total integrate intensity of pS20 MLC2 and F-actin staining in individual endothelial cells. For measuring subcortical levels: Integrate density from ROIs A2 to ROI A3 was calculated in individual endothelial cells. The mean of the integrate density of four cells per image, and at least four images per genotype and treatment were used for the quantification. β-catenin junctional positive area/cell perimeter was calculated per individual cell. For that, a manual threshold was set to obtain binary images. Then, total β-catenin area—β-catenin nuclear area in individual cells was calculated to obtain β-catenin junctional positive area. Junctional staining of β-catenin was used to measure the perimeter of individual cells. At least four cells per image and four images per genotype were used for the quantification. To measure the type of junctional coverage, percentage of junctional pattern was considered. ≥60% serrated pattern/cell = serrated, ≥ 60% of straight junctional pattern/cell = straight, ≈50% of each pattern/cell = mixed. Five cells per image and at least five images per genotype were used for the quantification.

### Mass spectrometry

Primary endothelial cells were washed twice with cold PBS supplemented with 1 mM Na_3_VO_4_ and 1 mM NaF and lysed with 300 µl of urea buffer (8 M Urea in 20 mM in HEPES pH 8.0 supplemented with 1 mM Na_3_VO_4_, 1 mM NaF, 1 mM Na_4_P_2_O_7_ and 1 mM sodium β-glycerophosphate) for 30 min. All mass spectrometry solvents were prepared in LC-MS grade water (LGC Promochem, #SO-9368-B025). We used published methods for phosphoproteome analyses^32,65,66^. In brief, 250 µg of protein were reduced and alkylated by sequential incubation with 10 mM DTT and 16.6 mM iodoacetamide for 1 h. Urea concentration was diluted to 2 M with 20 mM HEPES (pH 8.0), and 80 µl of trypsin beads (50% slurry of TLCK-trypsin (Thermo-Fisher Scientific, #20230)) pre-conditioned with three washes of 20 mM HEPES (pH 8.0) were added to the samples, followed by incubating the tubes for 16 h at 37 °C with agitation. Trypsin beads were removed by centrifugation at 2000 × *g* for 5 min at 5 °C. Following trypsin digestion, peptide solutions were desalted using 10 mg OASIS-HLB cartridges (Waters, #186000383). Briefly, OASIS cartridges were accommodated in a vacuum manifold (−5 mmHg), activated with 1 ml ACN (LGC Promochem, #SO-9340-B025) and equilibrated with 1.5 ml of washing solution (1% ACN, 0.1% TFA (LGC Promochem, #SO-9668-B001)). After loading the samples, cartridges were washed with 1 ml of washing solution. Phosphopeptides were eluted with 500 µl of glycolic acid buffer 1 (1 M glycolic acid (Acros Organics, #154515000), 50% ACN, 5% TFA) and subjected to phosphoenrichment. Phosphopeptides were enriched using TiO_2_ (GL Sciences, #5020-75010). Sample volumes were normalized to 1 ml using glycolic acid buffer 2 (1 M glycolic acid, 80% ACN, 5% TFA), 50 µl of TiO_2_ beads (50% slurry in 1% TFA) were added to the peptide mixture, incubated for 5 min at RT with agitation and centrifuged for 30 s at 1500 × *g*. For each sample, 80% of the supernatant was transferred to fresh tubes and stored in ice and the remaining 20% used to resuspend the bead pellets that were loaded into an empty prewashed PE-filtered spin-tips (Glygen, #TF2EMT.96) and packed by centrifugation at 1500 × *g* for 3 min. After loading the remaining volume of the supernatant by centrifugation at 1500 × *g* for 3 min, spin tips were sequentially washed with 100 µl of glycolic acid buffer 2, ammonium acetate buffer (100 mM ammonium acetate in 25% ACN) and 10% ACN by RT centrifugation for 3 min at 1500 × *g*. For phosphopeptide recovery, additional 50 µl of 5% ammonia solution (LGC Promochem, HPA-0070-B010) followed by centrifugation for 5 min at 1500 × *g* was repeated four times. Eluents were snap frozen in dry ice, dried in a speed vac (RVC 2-25, Martin Christ Gefriertrocknungsanlagen GmbH, Osterode am Harz, Germany) and peptide pellets stored at −80 °C. Peptide pellets were resuspended in 12 µl of reconstitution buffer (20 fmol/µl enolase (Waters, #186002325) in 3% ACN, 0.1% TFA) and 5 µl were loaded onto an LC-MS/MS system consisting of a Dionex UltiMate 3000 RSLC directly coupled to an Orbitrap Q-Exactive Plus mass spectrometer (Thermo Fisher Scientific). Peptides were trapped in a μ-pre-column (Acclaim PepMap 100, Themo Fisher Scientific, #160454) and separated in an analytical column (Acclaim PepMap 100, Thermo Fisher Scientific, #164569) using A (3% ACN, 0.1% FA (Thermo Fisher Scientific, # F-1850-PB08)) and B (100% ACN, 0.1% FA) solutions as mobile phases. The following parameters were used: 3−23% B gradient for 120 min and a flow rate of 0.3 µl min^−1^. As they eluted from the nano-LC system, peptides were infused into the online connected Q-Exactive Plus system operating with a 2.1 s duty cycle. Acquisition of full scan survey spectra (m/z 375−1500) with a 70,000 FWHM resolution was followed by data-dependent acquisition in which the 20 most intense ions were selected for HCD (higher energy collisional dissociation) and MS/MS scanning (200−2000 m/z) with a resolution of 17,500 FWHM. A 30 s dynamic exclusion period was enabled with an exclusion list with 10 ppm mass window. Overall duty cycle generated chromatographic peaks of approximately 30 s at the base, which allowed the construction of extracted ion chromatograms (XICs) with at least ten data points.

### Peptide identification and quantification

Mascot Daemon 2.5.0 was used to automate peptide identification from MS data. Peak list files (MGFs) from RAW data were generated with Mascot Distiller v2.5.1.0 and searched into the Mascot search engine (v2.5) in order to match MS/MS data to theoretical peptide fragmentation data^67^. The searches were performed against the SwissProt Database (uniprot_sprot_2014_08.fasta) with an FDR of 1% (specific FDR for each phosphopeptide identification is included in Supplementary Data 1). A maximum of 2 trypsin missed cleavages and a mass tolerance of ±10 ppm for the MS scans and ± 25 mmu for the MS/MS scans were allowed. Carbamidomethyl Cys as fixed modification, and phosphorylation at Ser, Thr, and Tyr, PyroGlu on N-terminal Gln and oxidation of Met as variable modifications were considered. The accuracy of phosphosite location within the identified peptides was assessed using delta score values as described by Savitski et al.^68^. Delta scores for each phosphopeptide are reported in Supplementary Data 1. In-house developed software (Pescal) was used for label-free peptide quantification^69^. Pescal constructs extracted ion chromatograms (XIC) for each identified peptide and measures the area of the XICs for all the peptides identified across all samples. Thresholds for XIC generation were ±7 ppm and ±2 min m/z and retention time windows, respectively and undetectable peptides were given an intensity value of 0. Values of two technical replicates per sample were averaged and intensity values for each peptide were normalized to total sample intensity. Normalized quantitative data were used to calculate fold changes between groups and statistical significance (assessed by Student’s *t* test) when necessary. The construction of volcano plots and heatmaps was automated with a script generated in R software using the ggplot package. Venn diagrams were constructed using the software Venny (v2.1, http://bioinfogp.cnb.csic.es/tools/venny).

### Statistics

Data were analysed using GraphPad Prism software and were presented as mean ± SEM (error bars). Sample size and experimental replicates were indicated in figure legends. Statistical analysis was performed by the nonparametric Mann−Whitney’s test or the parametric Student’s *t* test. ns not significant; *P < 0.05; **P < 0.01; ***P < 0.001; and ****P < 0.001 were considered statistically significant.

### Code availability

The mass spectrometry data is deposited in the PRIDE repository (www.ebi.ac.uk/pride/archive/) with the dataset identifier PXD007060. The codes used for analysing the mass spectrometry data are annotated in Supplementary Table 2.

### Reporting Summary

Further information on research design is available in the Nature Research Reporting Summary linked to this article.

## Electronic supplementary material

Supplementary Information

Description of Additional Supplementary Files

Supplementary Movie 1

Supplementary Movie 2

Supplementary Movie 3

Supplementary Movie 4

Supplementary Movie 5

Supplementary Movie 6

Supplementary Data 1

Reporting Summary

## Data Availability

The mass spectrometry data that support the findings of this study have been deposited and are publicly available at the ProteomeXchange Consortium via the PRIDE^70^ partner repository (www.ebi.ac.uk/pride/archive/) with the dataset identifier PXD007060. The rest of the data and materials generated within this study are available from the corresponding author upon request. A reporting summary for this article is available as a Supplementary Information file.
