# Supplementary material for: Endothelial cell rearrangements during vascular patterning require PI3-kinase-mediated inhibition of actomyosin contractility
Source: Nat Commun. 2018 Nov 16;9:4826. doi: 10.1038/s41467-018-07172-3 (PMC6240100; doi:10.1038/s41467-018-07172-3)
Supplement: Supplementary file 1 — Supplementary Information [file 41467_2018_7172_MOESM1_ESM.pdf]

Supplementary Information

Angulo-Urarte et al.

Endothelial cell rearrangements during vascular patterning require
PI3-kinase-mediated inhibition of actomyosin contractility

Supplementary Figure 1

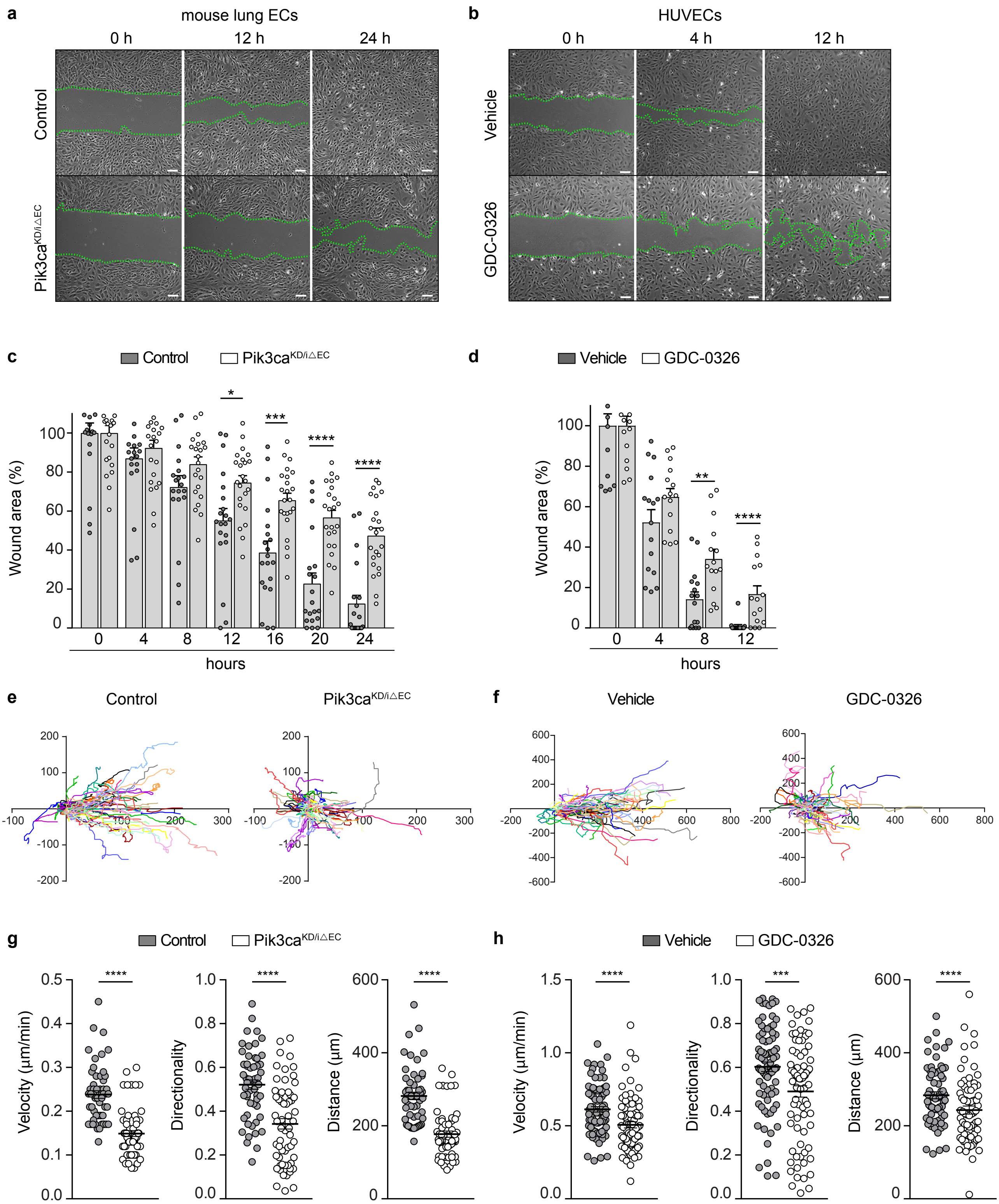

Supplementary Figure 1. Inactivation of PI3Kα impairs collective cell migration. (**a**) Representative phase contrast images of primary mouse endothelial cells isolated from control (top) and *Pik3ca$^{KD/i\Delta EC}$* (bottom) mice immediately after performing the scratch, and 12 h and 24 h later. Cells were treated with 4-OHT for 96 h before performing the scratch. (**b**) Representative phase contrast images of vehicle (DMSO, top) and GDC-0326 (1 µM, bottom) -treated HUVECs immediately after performing the scratch, and 4 h and 12 h later. Cells were treated with DMSO or GDC-0326 for 24 h before performing the scratch. Green dashed lines highlight open scratch-area in **a** and **b**. (**c, d**) Quantification of wound closure area at the indicated time-points after the scratch. n≥ 20 scratch areas per condition from three independent experiments in **c**, and n≥ 15 scratch areas per treatment from two independent experiments in **d**. (**e**, **f**) Plots depicting cell migration of control and *Pik3ca$^{KD/i\Delta EC}$* primary mouse endothelial cells during 20 h in a wound healing assay (**e**), and during 8 h in vehicle and GDC-0326 treated HUVECs (**f**). X and Y axis in the plots represent distance in µm. (**g**, **h**) Mean velocity directionality and travelled distance of individual cells measured during 20 h in **g** and 8 h in **h** after performing the scratch. n≥ 55 cells per condition from three independent experiments in **g,** and n≥ 104 cells per treatment from three independent experiments in **h**. Scale bars, 100 µm (**a, b**). Data in **c, d, g,** and **h** represent mean ± S.E.M. (error bars). *P<0.05, **P<0.01, ***P<0.001, and ****P<0.0001. Statistical analysis was performed with the two-sided Mann–Whitney test.

Supplementary Figure 2

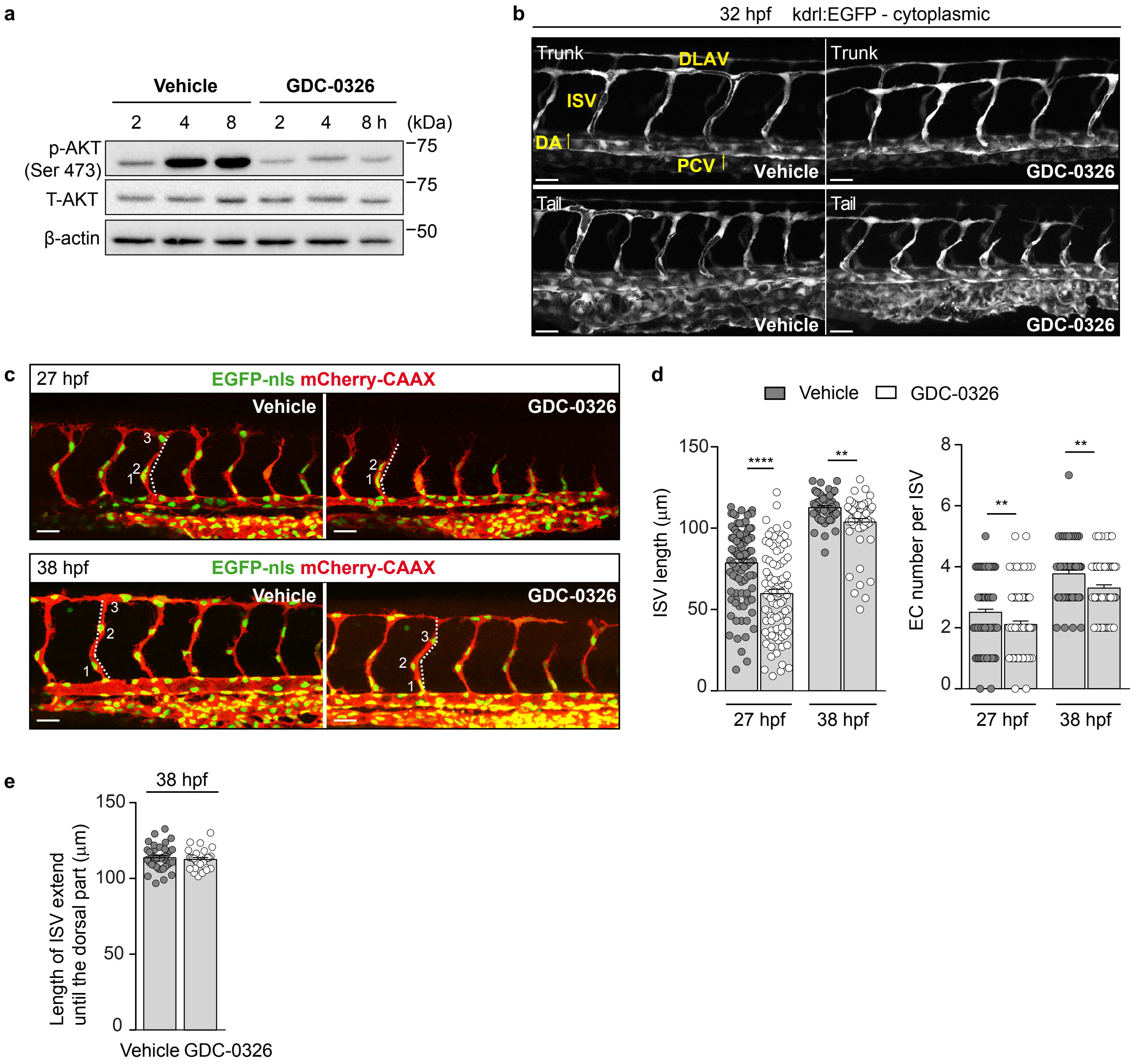

Supplementary Figure 2. Inactivation of PI3Kα in zebrafish embryos. (**a**) Wild type sibling zebrafish embryos were treated with vehicle (DMSO) or GDC-0326 (50 μM) at 22 hpf for 2 h, 4 h and 8 h followed by immunoblotting with the indicated antibodies. (**b**) Representative images of vehicle and GDC-0326 (50 μM) treated Tg(*kdrl:EGFP*)^{s843} zebrafish embryos from 22 to 32 hpf. DA refers to dorsal aorta, PCV refers to posterior cardinal vein, ISV refers to intersomitic vessels, and DLAV refers to dorsal longitudinal anastomotic vessels. (**c**) Representative images of vehicle and GDC-0326 (50 μM) treated Tg(*kdrl:EGFP-nls*)^{ubs1}/(*kdrl:mCherry-CAAX*)^{s916} transgenic embryos from 22 to 27 hpf (upper panel) or to 38 hpf (lower panel). ISV length (white punctuated lines) and nucleus number per ISV measurements are exemplified. Numbers indicate endothelial cell nuclei. (**d**) Quantitative analysis of vessel length and number of endothelial cells per ISV in zebrafish embryos shown in **c** (n≥ 52 ISVs per treatment). (**e**) Quantitative analysis of ISV length from the ISVs that have reached the top roof and started to form the DLAV at 38 hpf. Scale bars, 30 μm (**b, c**). Data in **d** and **e** represent mean ± S.E.M. (error bars). **P<0.01 and ****P<0.0001. Statistical analysis was performed with the two-sided Mann–Whitney test.

Supplementary Figure 3

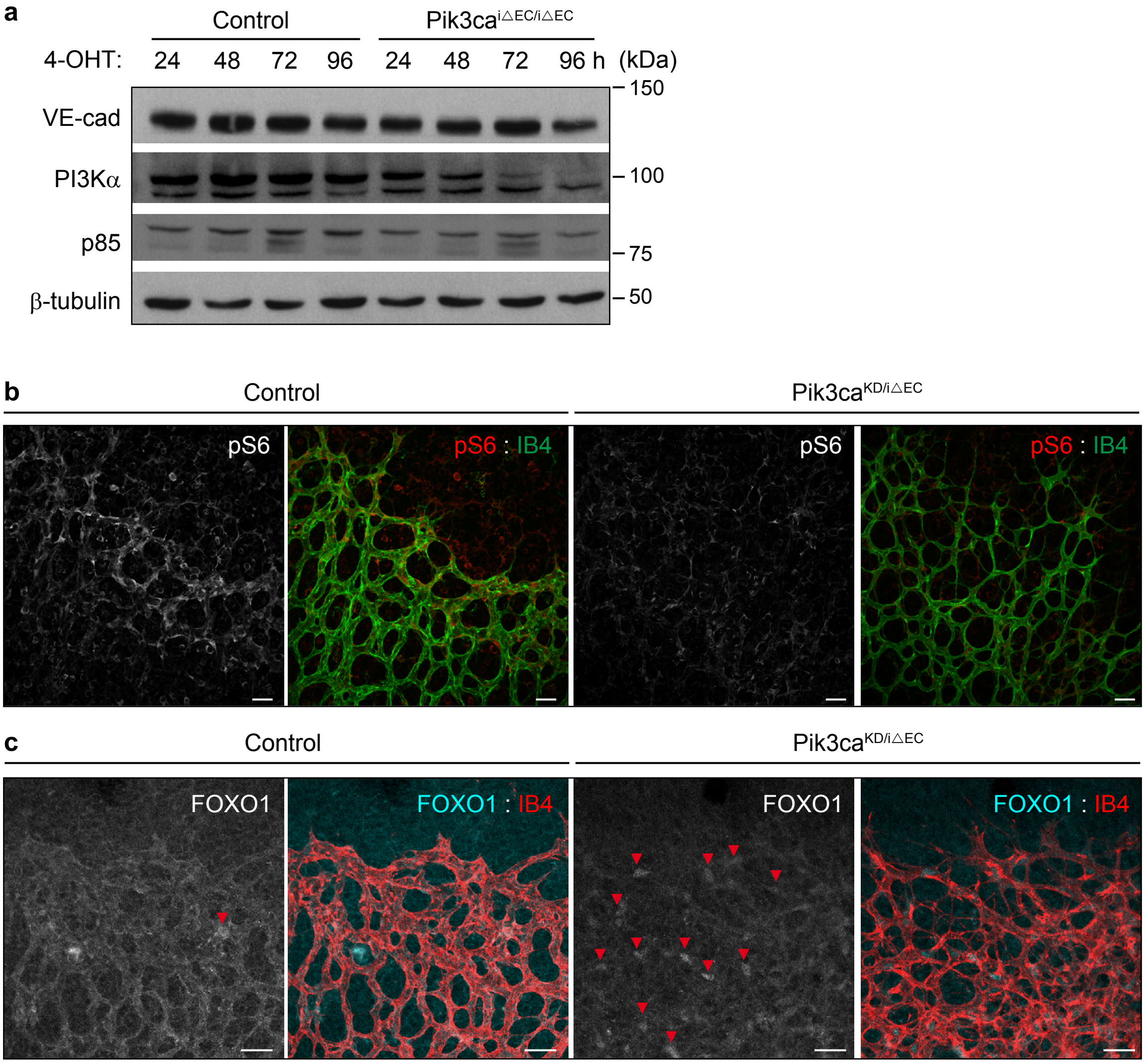

Supplementary Figure 3. Inactivation of PI3Kα in the mouse retinas. (**a**) Control and *Pik3ca*$^{i\Delta EC/i\Delta EC}$ mouse lung endothelial cells were incubated with 4-OHT for 24 h, 48 h, 72 h or 96 h, followed by immunoblotting using the indicated antibodies. (**b**) Representative images of pS6 240/242 (red) and IB4 (green)-stained P7 retinas of control and *Pik3ca*$^{KD/i\Delta EC}$ genotypes. (**c**) Confocal images of FOXO1 (cyan) and IB4 (red)-stained P7 retinas of control and *Pik3ca*$^{KD/i\Delta EC}$ genotypes. Scale bars, 30 µm (**b, c**).

Supplementary Figure 4

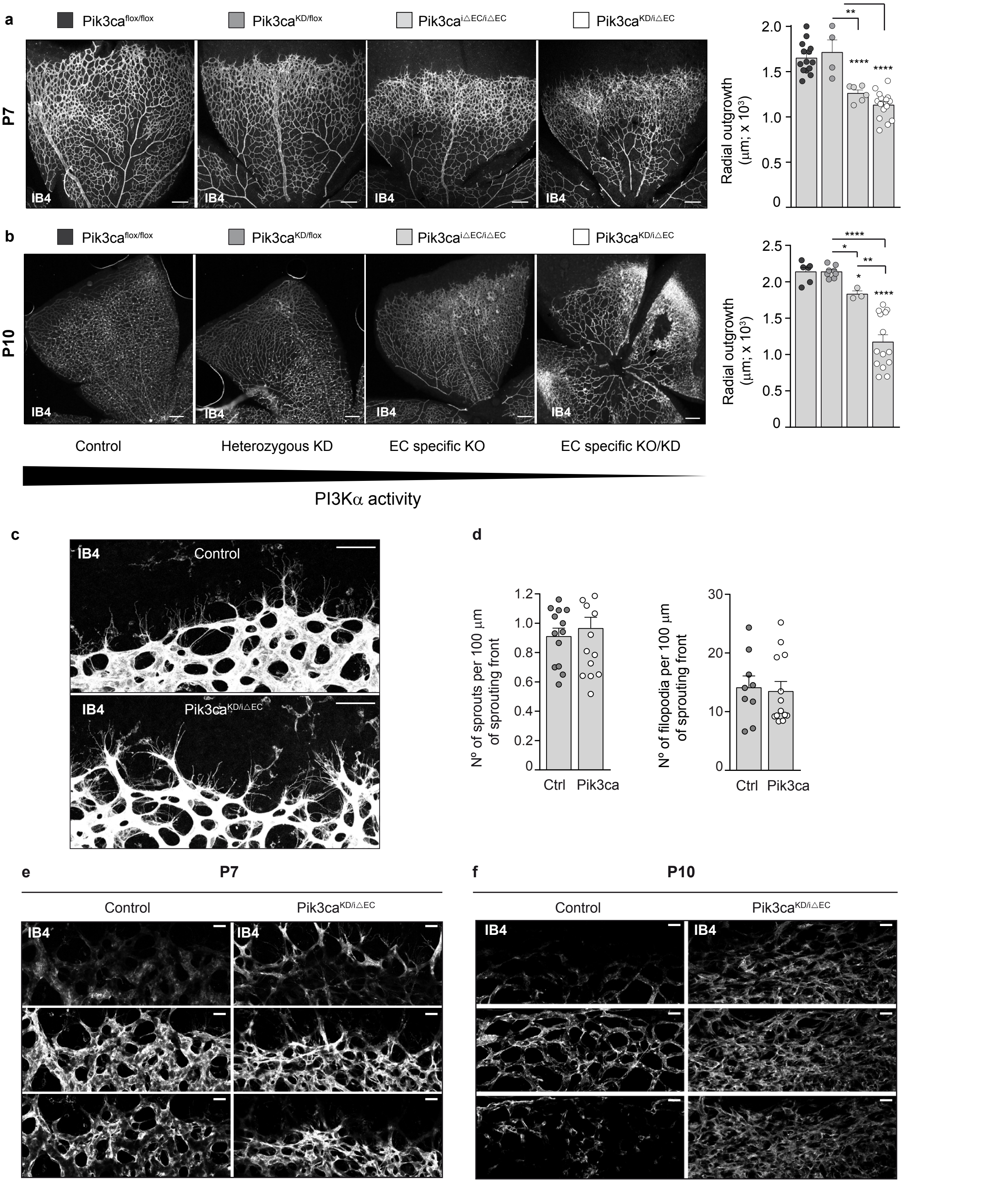

Supplementary Figure 4. Endothelial inactivation of PI3Kα leads to abnormal vessel growth. (**a**) Whole mount visualization of blood vessels by IB4 staining of P7 retinas from control, *Pik3ca$^{KD/flox}$*, *Pik3ca$^{\Delta EC/i\Delta EC}$* and *Pik3ca$^{KD/i\Delta EC}$* mouse littermates injected with 4-OHT at P1 and P2. Bars to the right show quantification of radial expansion of the vascular plexus in P7 retinas of respective genotypes. n≥ 4 retinas per genotype. (**b**) Whole mount visualization of blood vessels by IB4 staining of P10 retinas from control, *Pik3ca$^{KD/flox}$*, *Pik3ca$^{\Delta EC/i\Delta EC}$*, and *Pik3ca$^{KD/i\Delta EC}$* mouse littermates injected with 4-OHT at P1 and P2. Bars to the right show quantification of the radial expansion of the vascular plexus in P10 retinas of respective genotypes. n≥ 3 retinas per genotype. (**c**) Whole mount visualization of blood vessels by IB4 staining of control and *Pik3ca$^{KD/i\Delta EC}$* retinas at P7. (**d**) Quantitative analysis of number of sprouts (n≥ 13 retinas per genotype) and filopodial extensions (n≥ 9 retinas per genotype) of images shown in **c**. (**e**, **f**) Representative images corresponding to the top, middle and bottom layer of control and *Pik3ca$^{KD/i\Delta EC}$* P7 (**e**) and P10 (**f**) retinas visualized by IB4 staining. The same number and width of Z-stacks was taken per retina of each genotype. Scale bars, 150 μm (**a**), 200 μm (**b**), 50 μm (**c**), 20 μm (**e, f**). Data in **a, b**, and **d** represent mean ± S.E.M. (error bars). *P<0.05, **P<0.01, ***P<0.001, and ****P<0.0001. Statistical analysis was performed with the two-sided Mann–Whitney test.

Supplementary Figure 5

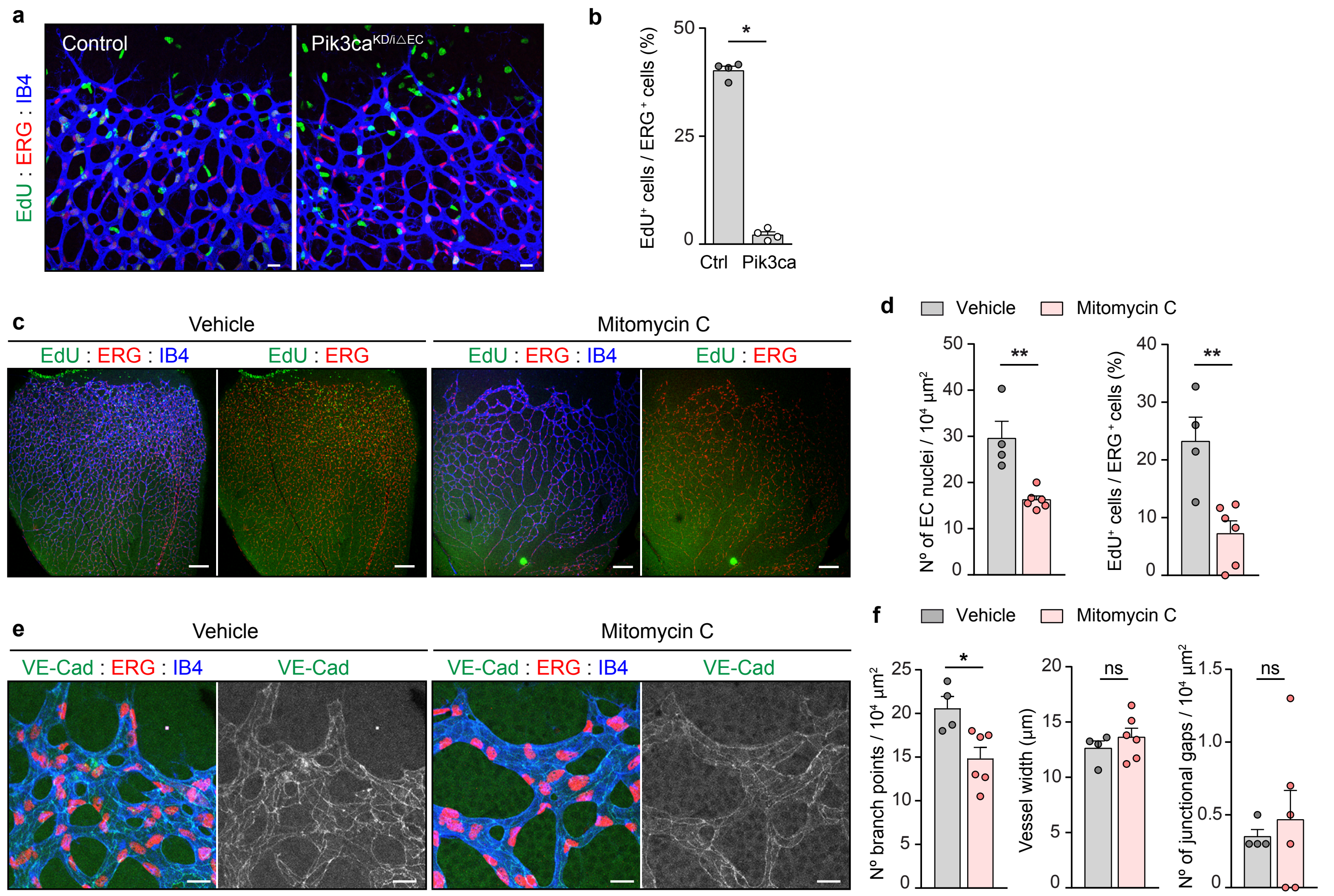

Supplementary Figure 5. Reduced proliferation does not interfere with junctional pattern in postnatal retinas. (**a**) EdU (green), ERG (red) and IB4 (blue) staining of control and *Pik3ca*$^{KD/i\Delta EC}$ P7 retinas. (**b**) Quantification of EdU-positive endothelial cells per total number of endothelial cells in control and *Pik3ca*$^{KD/i\Delta EC}$ P7 retinas (n≥4 retinas per genotype). (**c**) EdU (green), ERG (red) and IB4 (blue) staining of whole mount retinas from wild type mouse littermates treated with vehicle (DMSO) or mitomycin C at P5, and isolated at P7. (**d**) Quantification of endothelial cells per unit area assessed by ERG positivity (left panel) and of EdU positive endothelial cells per total number of endothelial cells (n≥ 4 retinas per treatment). (**e**) Representative images of whole mount retinas stained for VE-cadherin (green), ERG (red) and IB4 (blue) from wild type mouse littermates treated with vehicle or mitomycin C at P5 and isolated at P7. (**f**) Quantification of branch points, vessel width, and number of junctional gaps per unit area of images shown in **e** (n≥ 6 retinas per treatment). Scale bars, 20 µm (**a, e**), 140 µm (**c**). Data in **d**, **f** represent mean ± S.E.M. (error bars). ns: not significant,*P<0.05, **P<0.01. Statistical analysis was performed with the two-sided Mann–Whitney test.

Supplementary Figure 6

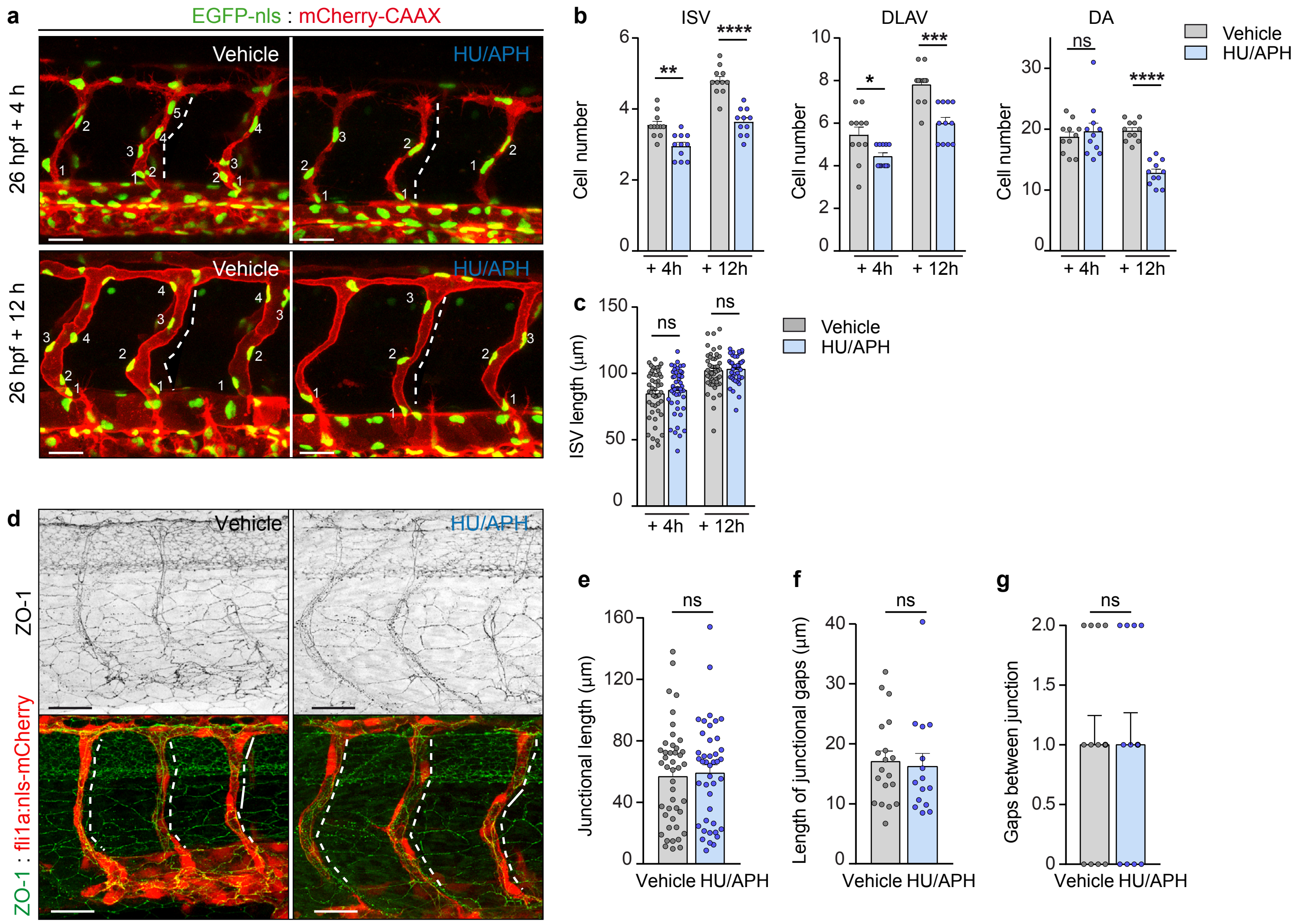

Supplementary Figure 6. Reduced proliferation does not interfere with junctional pattern in zebrafish embryos. (**a**) Images of vehicle and HU/APH-treated Tg(*kdrl:EGFP-nls*)^{ubs1}/(*kdrl:mCherry-CAAX*)^{s916} transgenic embryos from 26 hpf + 4 h (treatment) (upper panel) or to 26 hpf + 12 h treatment (lower panel). ISV length (white punctuated lines) and nucleus number per ISV measurements are exemplified. Number indicates endothelial cell nuclei. (**b**) Analysis of number of endothelial cells per ISV, DLAV and dorsal aorta (within 5 ISVs) in vehicle and HU/APH-treated zebrafish embryos (n=11 embryos per treatment, 5 ISVs per embryo, three independent experiments). (**c**) Analysis of ISV length (n≥42 ISVs per treatment, from 11-12 embryos analyzed from three independent experiments) in vehicle and HU/APH-treated zebrafish embryos. (**d**) Lateral views of intersomitic vessels (ISV) in vehicle or HU/APH treated transgenic Tg(*fli1ep:mCherry-nls*)^{ubs10} (shown in red) embryos stained for ZO-1 (green) at 33-34 hpf. Single ZO-1 staining is shown in row 1. White lines indicate ZO-1 negative staining (junctional gaps); white punctuate lines indicate elongation of junction. (**e**) Quantification of the length of the ISVs with continuous ZO-1 staining (n≥42 ISVs per treatment, from 11-12 embryos analyzed from three independent experiments). (**f**) Quantification of the length of the dorsal part of the ISVs without ZO-1 (length of junctional gaps) (ISVs without junctional gaps were excluded, n≥15 ISVs per treatment, from 11-12 embryos analyzed from three independent experiments). (**g**) Average number of gaps per ISV in vehicle and HU/APH-treated embryos (n=11 embryos per treatment, 5 ISVs per embryo, three independent experiments). Scale bars, 30 μm (**a, d**). Data in **b**, **c**, **e**, **f** and **g** represent mean ± S.E.M. (error bars). ns: not significant, **P<0.01, ***P<0.001, ****P<0.0001. Statistical analysis was performed with the two-sided Mann–Whitney test.

Supplementary Figure 7

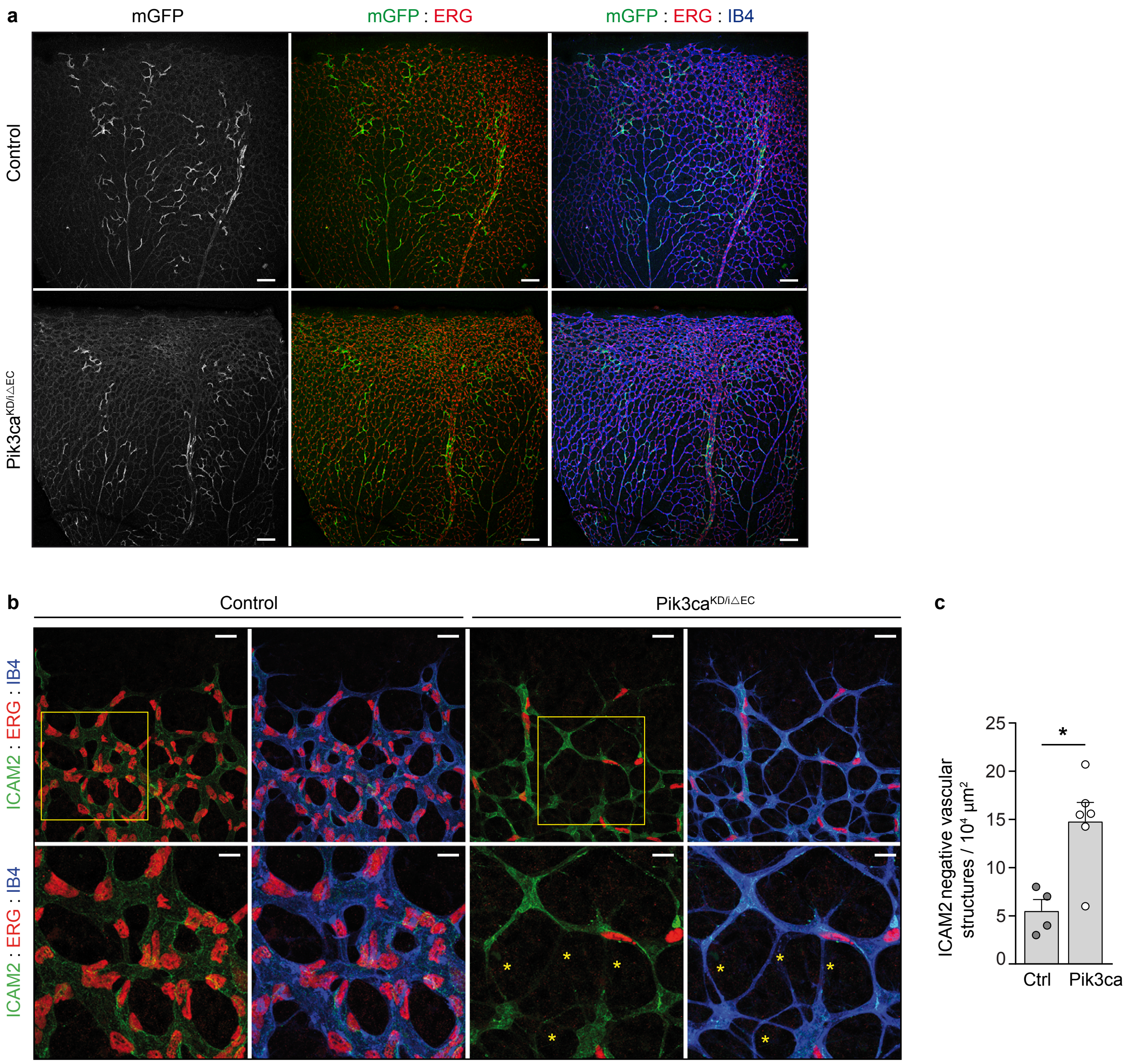

Supplementary Figure 7. Aberrant shape and lumen formation in *Pik3ca$^{KD/i\Delta EC}$* endothelium. (**a**) Overview of immunostaining of single-cell labelling using Cre-induced expression of membrane-bound GFP (mGFP, shown in green in the image), endothelial nuclei (ERG, shown in red) and blood vessel (IB4, shown in blue). (**b**) Control and *Pik3ca$^{KD/i\Delta EC}$* P7 retinas showing ICAM2 (green), ERG (red), and IB4 (blue). Yellow asterisks indicate IB4 positive/ICAM2 negative protrusions. (**c**) Quantification of ICAM2 negative vessels per unit area of images shown in **b** (n=4 retinas per genotype). Scale bars, 100 µm (**a**), 40 µm (**b**, upper panel), 10 µm (**b**, lower panel). Data represent mean ± S.E.M. (error bars). *P<0.05. Statistical analysis was performed with the two-sided Mann–Whitney test.

Supplementary Figure 8

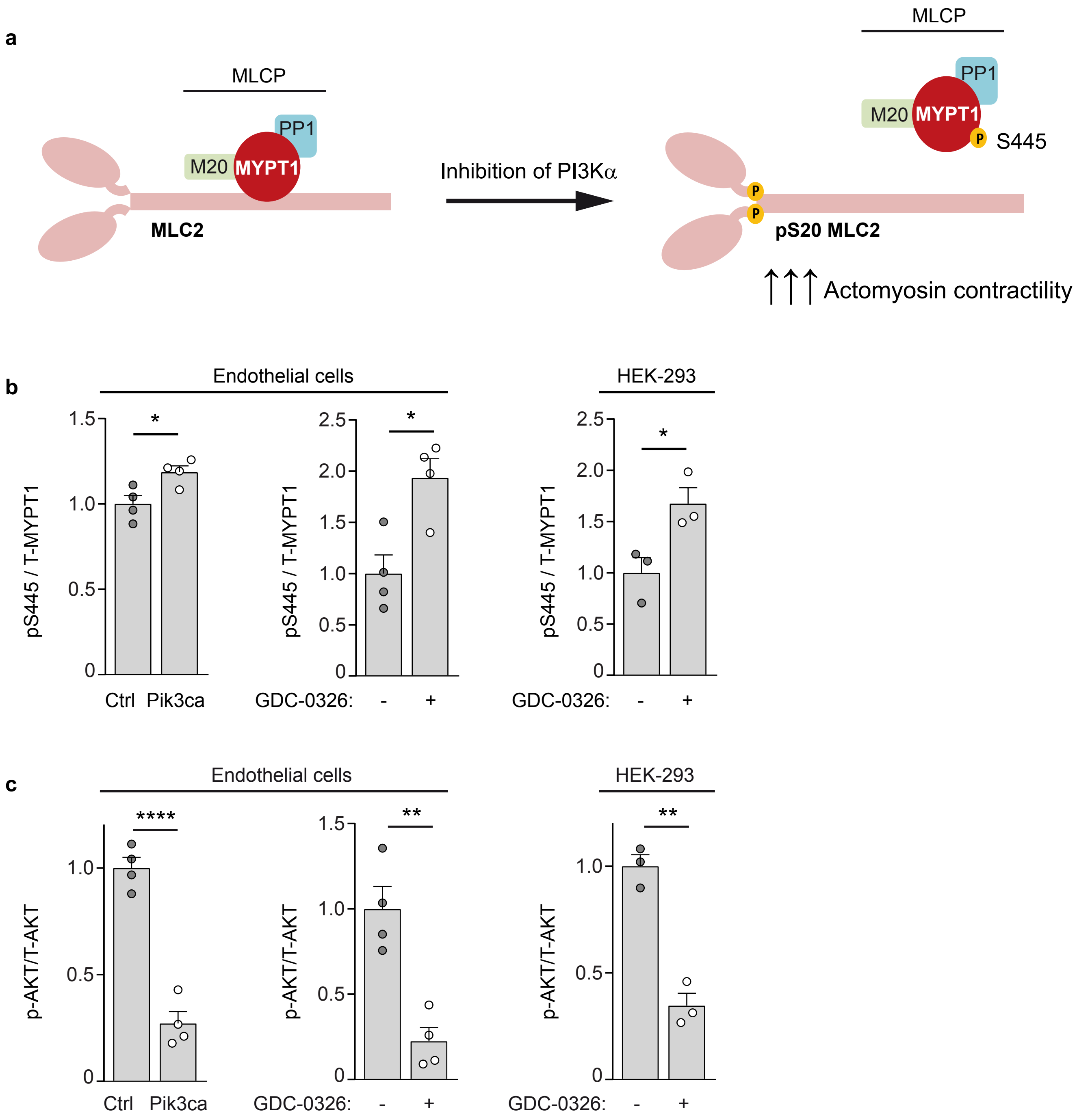

Supplementary Figure 8. Analysis of phosphoproteomic screening. (**a**) Schematic representation of the expected impact of inhibition of PI3Kα signalling on pS20 MLC2 and actomyosin contractility. MLCP is a trimeric heteroenzyme (PP1 catalytic subunit, MYPT1 regulatory subunit, and M20 subunit) that inactivates non-muscle myosin II by the dephosphorylation of MLC2. We observed that inhibition of PI3Kα results in phosphorylation of MYPT1 on the S445 residue favouring the delocalization of the MLCP complex from its substrate leading to an increase in the phosphorylation of MLC2 on S20, and as result, an increase in actomyosin contractility. Designed by Piotr Kobialka and Mariona Graupera. (**b, c**) Quantification of the relative immunoreactivity of pS445 MYPT1 normalized to total MYPT1 (**b**) and of pS473 AKT normalized to total AKT (**c**) from western blots shown in Fig. 4e and represented as the mean of at least three independent experiments. Data represent mean ± S.E.M. (error bars). *P<0.05, **P<0.01, and ****P<0.0001. Statistical analysis was performed by the two-sided Student´s t test.

Supplementary Figure 9

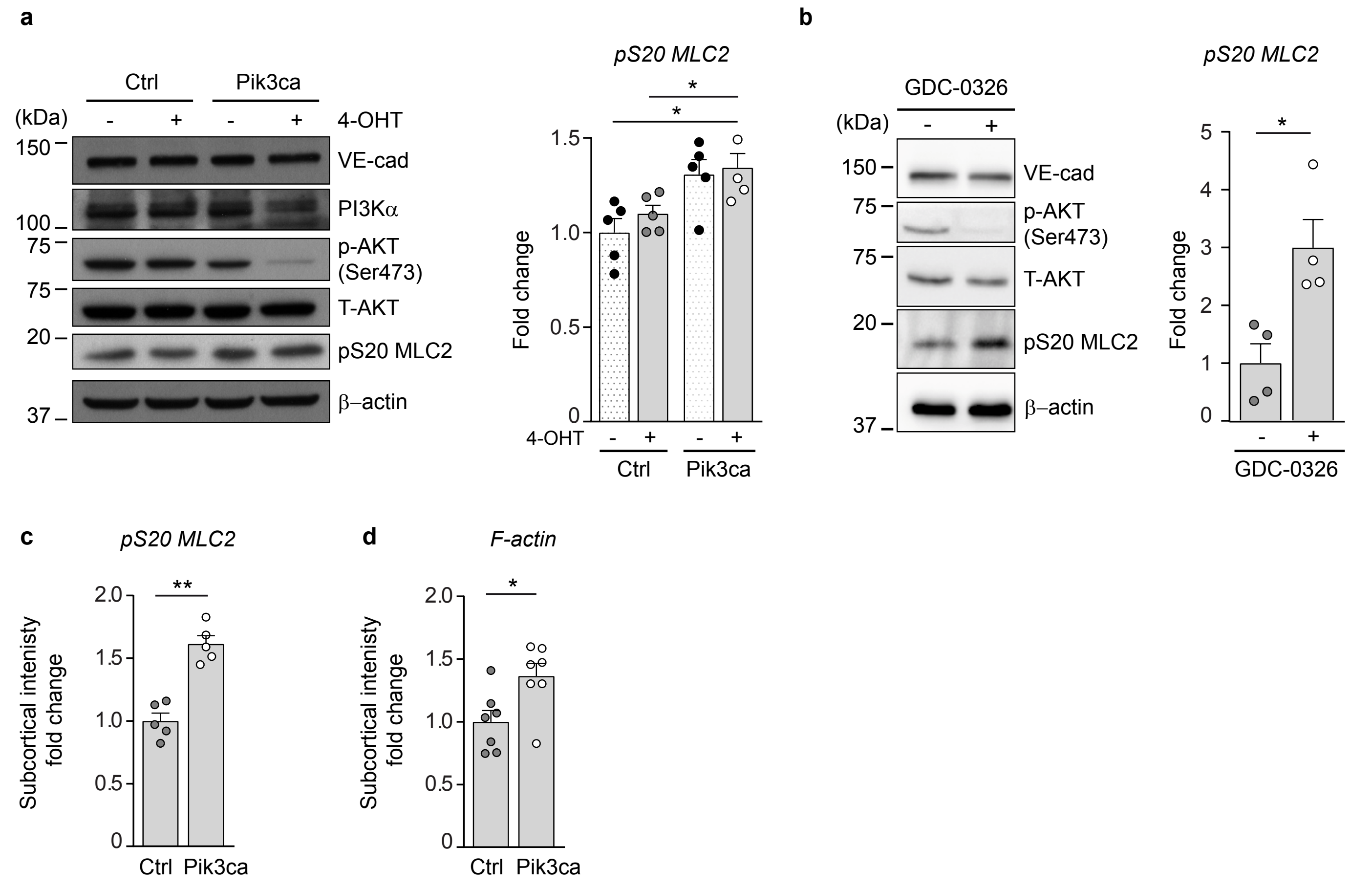

Supplementary Figure 9. Impact of inactivation of PI3Kα on pS20 MLC2 and its localization in endothelial cells. (**a**) Control and *Pik3ca*$^{KD/i\Delta EC}$ endothelial cells treated with 4-OHT for 96 h, followed by immunoblotting using the indicated antibodies. Quantification of pS20 MLC2 is shown to the right (n=5 independent experiments). (**b**) Wild type mouse lung endothelial cells were incubated with vehicle (DMSO) or GDC-0326 (1 μm) for 48 h followed by immunoblotting using the indicated antibodies. Quantification of pS20 MLC2 is shown to the right (n=4 independent experiments). (**c**, **d**) Quantification of pS20 MLC2 (**c**) and F-actin (**d**) immunostaining intensity at the subcortical location (shown as integrated density) of control and *Pik3ca*$^{KD/i\Delta EC}$ endothelial cells treated with 4-OHT for 96 h (n≥5 independent experiments). Data in **a, b, c** and **d** represent mean ± S.E.M. (error bars). *P<0.05 and **P<0.01. Statistical analysis was performed with the two-sided Mann–Whitney test.

Supplementary Figure 10

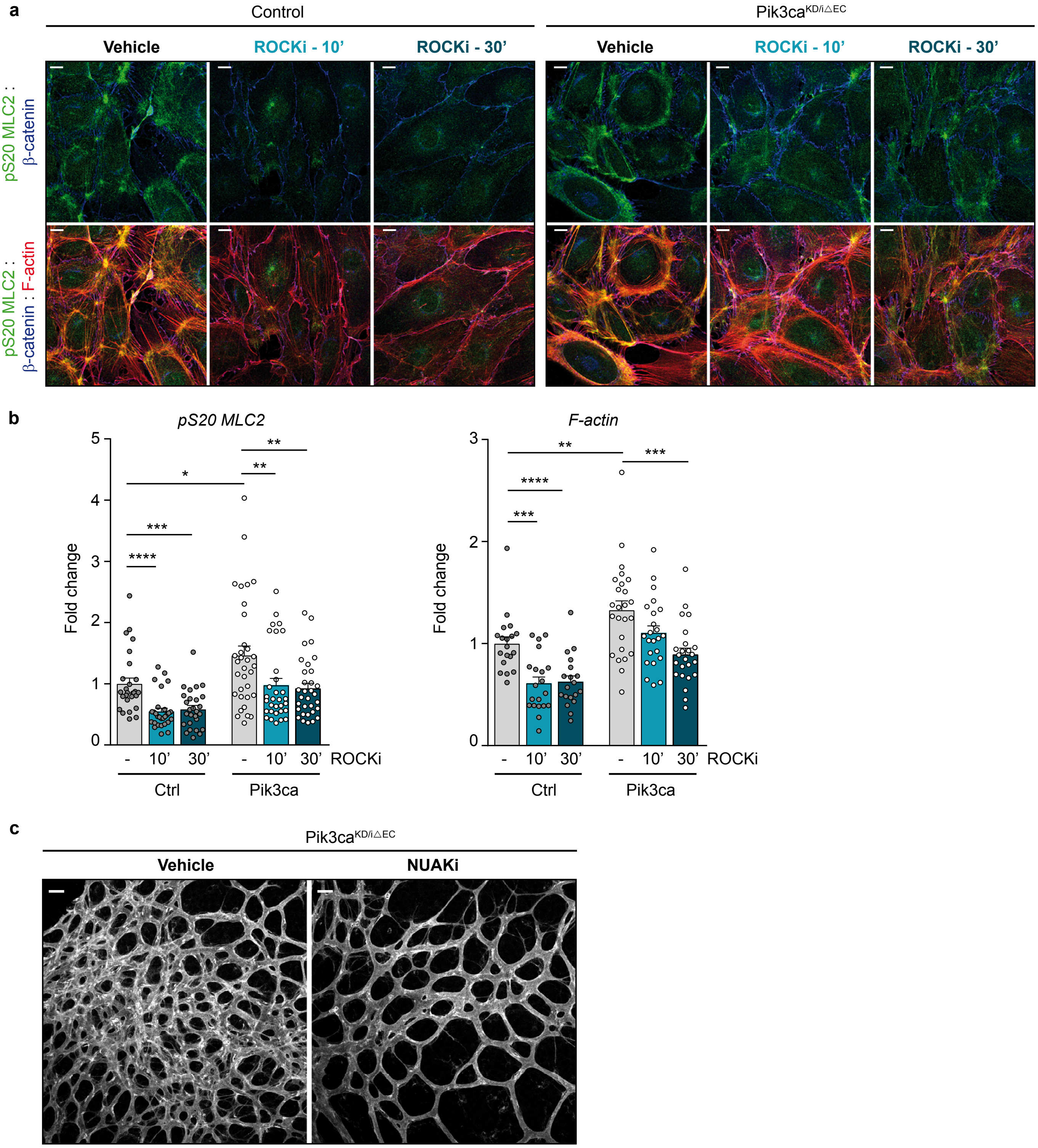

This figure continues in the next page.

Supplementary Figure 10 extension

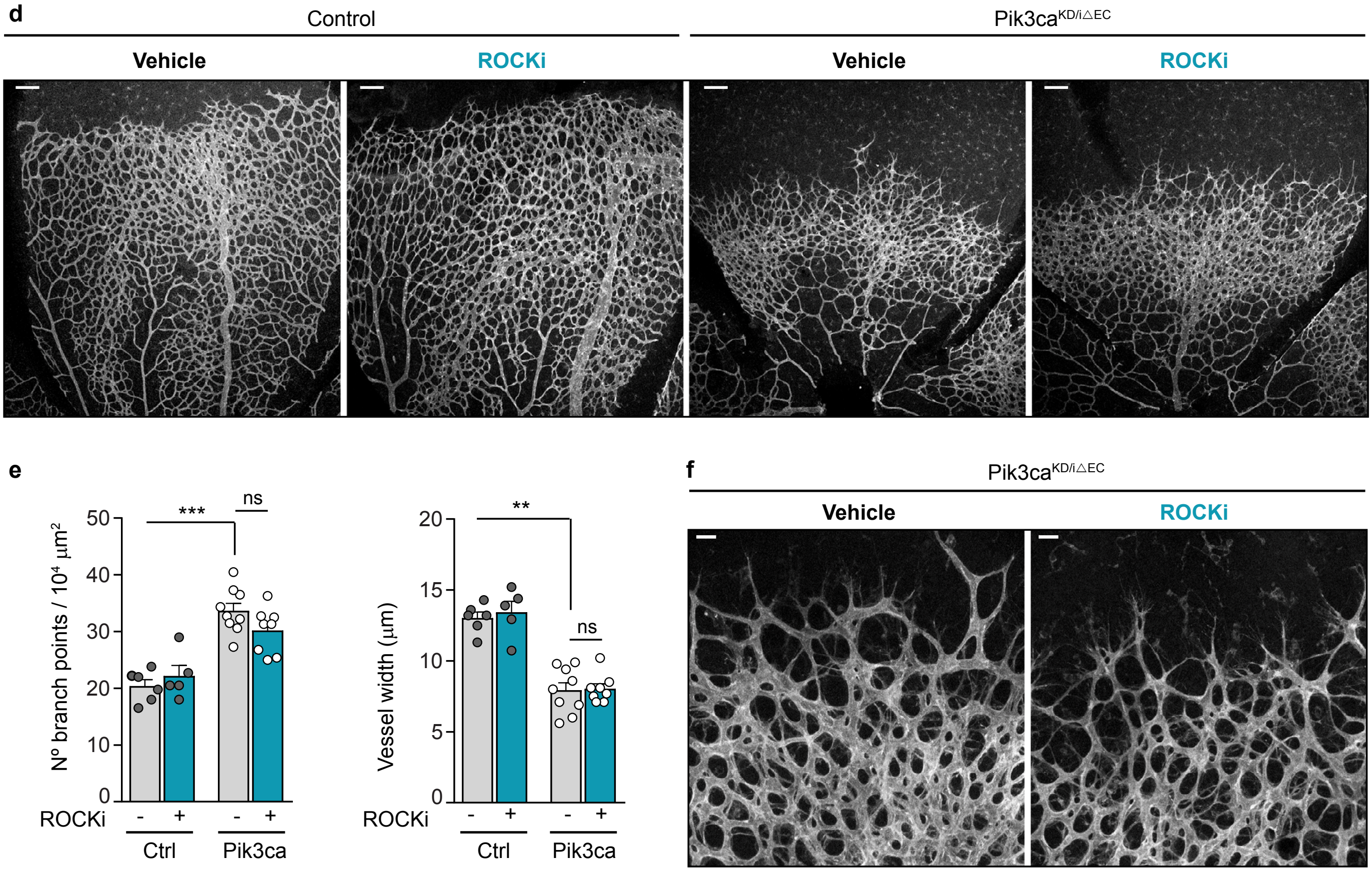

Supplementary Figure 10. Inactivation of ROCK does not restore the endothelial phenotypes imposed by PI3Kα inactivation. (**a**) Assessment of pS20 MLC2 (green), F-actin (red), and β-catenin (blue) of control and *Pik3ca*$^{KD/i\Delta EC}$ endothelial cells treated with 4-OHT for 96 h and incubated with DMSO as control or 10 µM Y-27632 (ROCK inhibitor; ROCKi) for 10 or 30 min. (**b**) Subcortical quantification of pS20 MLC2 (left panel) and F-actin (right panel) immunostaining intensities (shown as integrated density) of images shown in **a**. (n≥ 28 images of at least three independent experiments). (**c**) High magnification confocal images of *Pik3ca*$^{KD/i\Delta EC}$ P7 retinas treated with DMSO or WZ4003 (NUAKi) at P6. (**d**) IB4-stained control and *Pik3ca*$^{KD/i\Delta EC}$ P7 retinas treated with DMSO or Y-27632 (ROCKi) at P6 and P7 (10 am). (**e**) Quantification of branch points per unit area and vessel width per unit area (n≥5 retinas per genotype and treatment). (**f**) High magnification confocal images of *Pik3ca*$^{KD/i\Delta EC}$ P7 retinas treated with DMSO or Y-27632. Scale bars, 15 µm (**a**), 20 µm (**c**), 100 µm (**d**), 20 µm (**f**). Data in **b** and **e** represent mean ± S.E.M. (error bars). ns: not significant, *P<0.05, **P<0.01, ***P<0.001, and ****P<0.0001. Statistical analysis was performed with the two-sided Mann–Whitney test.

Supplementary Figure 11

a Supplementary Figure 2a

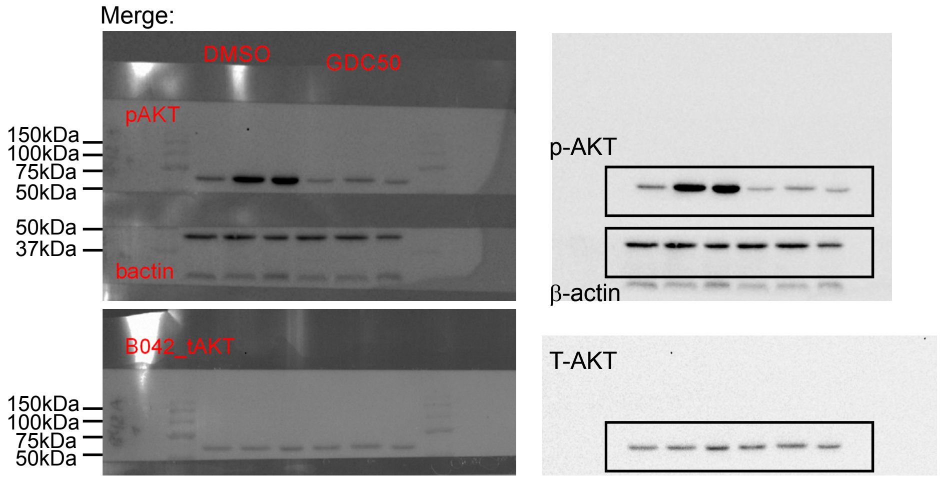

b Supplementary Figure 3a

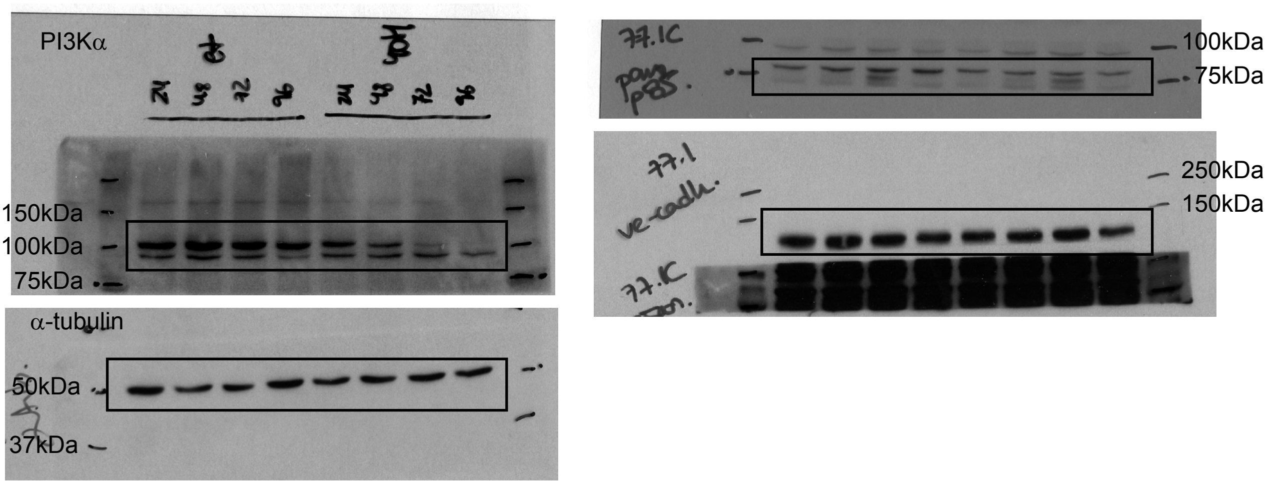

Supplementary Figure 11. Uncropped western blots. (**a**) Blots related to Supplementary Figure 2a. Left panels show the merge with the molecular markers and the right panels show the boxes indicating the cropped sections used in the corresponding figures. (**b**) Blots related to Supplementary Figure 3a. Boxes indicate the cropped sections used in the corresponding figures.

Of note, for some immunoblotting assays membranes were cut into several pieces to incubate with different antibodies.

Supplementary Figure 12

a Figure 4e

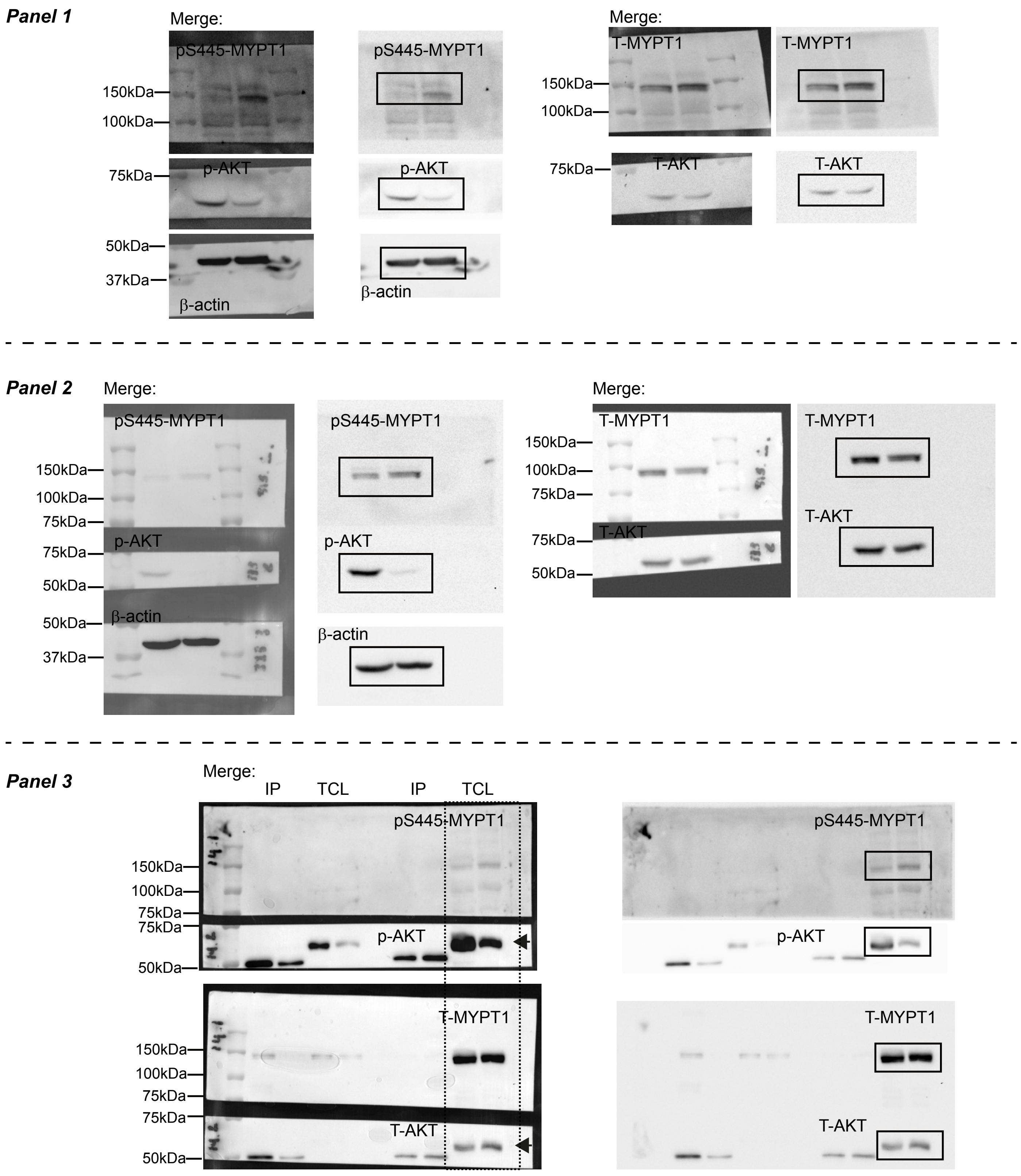

Supplementary Figure 12. Uncropped western blots. Blots related to Figure 4e. Left panels show the merge with the molecular markers and the right panels show the boxes indicating the cropped sections used in the corresponding figures. Of note, for some immunoblotting assays membranes were cut into several pieces to incubate with different antibodies.

Supplementary Figure 13

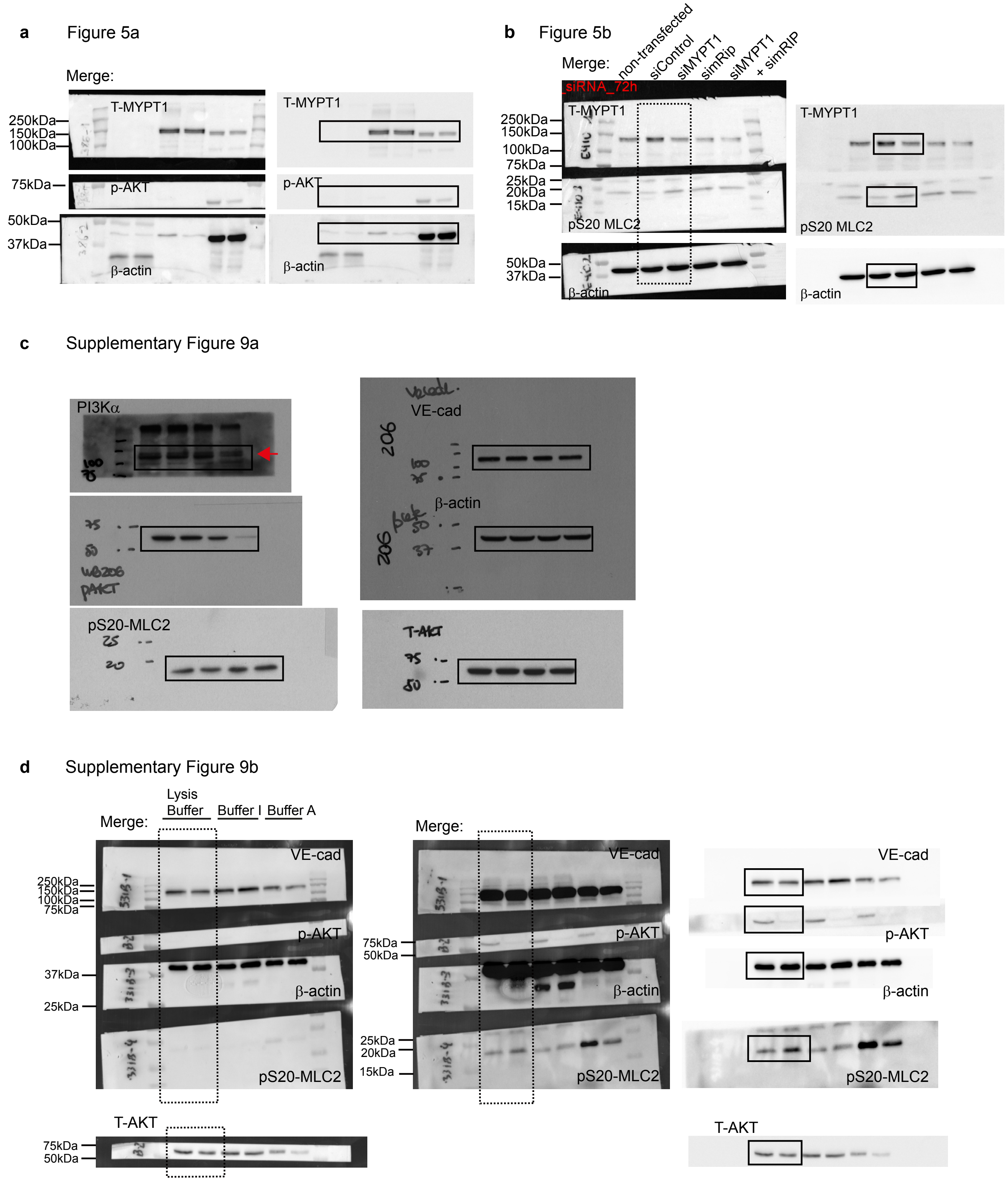

Supplementary Figure 13. Uncropped western blots. (**a**) Blots related to Figure 5a. (**b**) Blots related to Figure 5b. (**c**) Blots related to Supplementary Figure 9a. (**d**) Blots related to Supplementary Figure 9b. Left panels show the merge with the molecular markers and the right panels show the boxes indicating the cropped sections used in the corresponding figures. Of note, for some immunoblotting assays membranes were cut into several pieces to incubate with different antibodies.

Supplementary Table 1. Summary of genetic models use to study *Pik3ca* inactivation

Mouse model	Name after 4-OHT treatment	Description
Pik3ca$^{flox/flox}$	Control	Control. No Cre recombinase expression
Pik3ca$^{KD/flox}$	*Pik3ca*$^{KD/flox}$	Heterozygous kinase dead (KD). One *Pik3ca* alelle expresses constitutively a kinase dead form of the PI3Kα protein. Half of the PI3Kα protein is inactive.
Pdgfb-iCreER Pik3ca$^{flox/flox}$	*Pik3ca*$^{i\Delta EC/i\Delta EC}$	Endothelial specific knockout mouse (KO). Endothelial specific deletion of both *Pi3kca* alleles after 4-HOT administration
Pdgfb-iCreER Pik3ca$^{KD/flox}$	*Pik3ca*$^{KD/i\Delta EC}$	Kinase dead allele and endothelial specific knockout allele. One *Pik3ca* allele expresses constitutively a kinase dead form of the PI3Kα protein and the other is deleted after 4-OHT administration. Main model of study in this manuscript.

Supplementary Table 2. Codes used for the analysis of the mass spectrometry data.

Sample Name	Animal ID	Genotype	Treatment	Replicate
R_b1822p5_S10_SF622_24h_ETH_Phos_R1.raw	SF622	Control	24h ethanol	1
R_b1822p5_S10_SF622_24h_ETH_Phos_R2.raw	SF622	Control	24h ethanol	2
R_b1822p5_S11_SF622_24h_4HTA_Phos_R1.raw	SF622	Control	24h 4OHT	1
R_b1822p5_S11_SF622_24h_4HTA_Phos_R2.raw	SF622	Control	24h 4OHT	2
R_b1822p5_S12_SF622_96h_4HTA_Phos_R1.raw	SF622	Control	96h 4OHT	1
R_b1822p5_S12_SF622_96h_4HTA_Phos_R2.raw	SF622	Control	96h 4OHT	2
R_b1822p5_S13_SF602_24h_ETH_Phos_R1.raw	SF602	Control	24h ethanol	1
R_b1822p5_S13_SF602_24h_ETH_Phos_R2.raw	SF602	Control	24h ethanol	2
R_b1822p5_S14_SF602_24h_4HTA_Phos_R1.raw	SF602	Control	24h 4OHT	1
R_b1822p5_S14_SF602_24h_4HTA_Phos_R2.raw	SF602	Control	24h 4OHT	2
R_b1822p5_S15_SF602_96h_4HTA_Phos_R1.raw	SF602	Control	96h 4OHT	1
R_b1822p5_S15_SF602_96h_4HTA_Phos_R2.raw	SF602	Control	96h 4OHT	2
R_b1822p5_S16_SF603_24h_ETH_Phos_R1.raw	SF603	Control	24h ethanol	1
R_b1822p5_S16_SF603_24h_ETH_Phos_R2.raw	SF603	Control	24h ethanol	2
R_b1822p5_S17_SF603_24h_4HTA_Phos_R1.raw	SF603	Control	24h 4OHT	1
R_b1822p5_S17_SF603_24h_4HTA_Phos_R2.raw	SF603	Control	24h 4OHT	2
R_b1822p5_S18_SF603_96h_4HTA_Phos_R1.raw	SF603	Control	96h 4OHT	1
R_b1822p5_S18_SF603_96h_4HTA_Phos_R2.raw	SF603	Control	96h 4OHT	2
R_b1822p5_S19_SF604_24h_ETH_Phos_R1.raw	SF604	$Pik3CA^{KD/Flox}$	24h ethanol	1
R_b1822p5_S19_SF604_24h_ETH_Phos_R2.raw	SF604	$Pik3CA^{KD/Flox}$	24h ethanol	2
R_b1822p5_S1_SF619_24h_ETH_Phos_R1.raw	SF604	$Pik3CA^{KD/Flox}$	24h ethanol	1
R_b1822p5_S1_SF619_24h_ETH_Phos_R2.raw	SF604	$Pik3CA^{KD/Flox}$	24h ethanol	2
R_b1822p5_S20_SF604_24h_4HTA_Phos_R1.raw	SF604	$Pik3CA^{KD/Flox}$	24h 4OHT	1
R_b1822p5_S20_SF604_24h_4HTA_Phos_R2.raw	SF604	$Pik3CA^{KD/Flox}$	24h 4OHT	2
R_b1822p5_S21_SF604_96h_4HTA_Phos_R1.raw	SF604	$Pik3CA^{KD/Flox}$	96h 4OHT	1
R_b1822p5_S21_SF604_96h_4HTA_Phos_R2.raw	SF604	$Pik3CA^{KD/Flox}$	96h 4OHT	2
R_b1822p5_S22_SF607_24h_ETH_Phos_R1.raw	SF607	$Pik3CA^{KD/Flox}$	24h ethanol	1
R_b1822p5_S22_SF607_24h_ETH_Phos_R2.raw	SF607	$Pik3CA^{KD/Flox}$	24h ethanol	2
R_b1822p5_S23_SF607_24h_4HTA_Phos_R1.raw	SF607	$Pik3CA^{KD/Flox}$	24h 4OHT	1
R_b1822p5_S23_SF607_24h_4HTA_Phos_R2.raw	SF607	$Pik3CA^{KD/Flox}$	24h 4OHT	2
R_b1822p5_S24_SF607_96h_4HTA_Phos_R1.raw	SF607	$Pik3CA^{KD/Flox}$	96h 4OHT	1
R_b1822p5_S24_SF607_96h_4HTA_Phos_R2.raw	SF607	$Pik3CA^{KD/Flox}$	96h 4OHT	2
R_b1822p5_S2_SF619_24h_4HTA_Phos_R1.raw	SF619	$Pik3CA^{KD/Flox}$	24h 4OHT	1
R_b1822p5_S2_SF619_24h_4HTA_Phos_R2.raw	SF619	$Pik3CA^{KD/Flox}$	24h 4OHT	2
R_b1822p5_S3_SF619_96h_4HTA_Phos_R1.raw	SF619	$Pik3CA^{KD/Flox}$	96h 4OHT	1
R_b1822p5_S3_SF619_96h_4HTA_Phos_R2.raw	SF619	$Pik3CA^{KD/Flox}$	96h 4OHT	2
R_b1822p5_S4_SF624_24h_ETH_Phos_R1.raw	SF624	Control	24h ethanol	1
R_b1822p5_S4_SF624_24h_ETH_Phos_R2.raw	SF624	Control	24h ethanol	2
R_b1822p5_S5_SF624_24h_4HTA_Phos_R1.raw	SF624	Control	24h 4OHT	1
R_b1822p5_S5_SF624_24h_4HTA_Phos_R2.raw	SF624	Control	24h 4OHT	2
R_b1822p5_S6_SF624_96h_4HTA_Phos_R1.raw	SF624	Control	96h 4OHT	1
R_b1822p5_S6_SF624_96h_4HTA_Phos_R2.raw	SF624	Control	96h 4OHT	2
R_b1822p5_S7_SF621_24h_ETH_Phos_R1.raw	SF621	$Pik3CA^{KD/Flox}$	24h ethanol	1
R_b1822p5_S7_SF621_24h_ETH_Phos_R2.raw	SF621	$Pik3CA^{KD/Flox}$	24h ethanol	2
R_b1822p5_S8_SF621_24h_4HTA_Phos_R1.raw	SF621	$Pik3CA^{KD/Flox}$	24h 4OHT	1
R_b1822p5_S8_SF621_24h_4HTA_Phos_R2.raw	SF621	$Pik3CA^{KD/Flox}$	24h 4OHT	2
R_b1822p5_S9_SF621_96h_4HTA_Phos_R1.raw	SF621	$Pik3CA^{KD/Flox}$	96h 4OHT	1
R_b1822p5_S9_SF621_96h_4HTA_Phos_R2.raw	SF621	$Pik3CA^{KD/Flox}$	96h 4OHT	2
