# Supplementary material for: Endothelial cell rearrangements during vascular patterning require PI3-kinase-mediated inhibition of actomyosin contractility
Source: Nat Commun. 2018 Nov 16;9:4826. doi: 10.1038/s41467-018-07172-3 (PMC6240100; doi:10.1038/s41467-018-07172-3)
Supplement: Supplementary file 2 — Description of Additional Supplementary Files [file 41467_2018_7172_MOESM2_ESM.docx]

**Description of Additional Supplementary Files**

File Name: Supplementary Movie 1.

Description: Phase-contrast live imaging of scratch-wound closure of control and Pik3caKD/iΔEC mouse lung endothelial cell monolayers. A scratch assay showing control (left) and Pik3caKD/iΔEC (right) mouse lung endothelial cells. Images were acquired every 10 min.

File Name: Supplementary Movie 2.

Description: Phase-contrast live imaging of scratch-wound closure of vehicle and GDC-0326 treated HUVEC monolayers. A scratch assay showing vehicle (DMSO, left) or GDC0326 (right) treated HUVECs. Images were acquired every 10 min

File Name: Supplementary Movie 3.

Description: 3D reconstruction of a P7 control retina stained for IB4 to visualize blood vessels.

File Name: Supplementary Movie 4.

Description: 3D reconstruction of a P7 Pik3caKD/iΔEC retina stained for IB4 visualize blood vessels.

File Name: Supplementary Movie 5.

Description: Early anastomosis steps in control zebrafish embryos. Time-lapse series of the lateral view of a Tg(UAS:EGFP-UCHD) ubs18, Tg(kdrl:mCherry-CAAX) s916 embryo at around 30 hpf. Confocal stacks were acquired every 15 min. Endothelial cell membrane is visualized in red and actin cytoskeleton is visualized by F-actin binding domain of utrophin in green.

File Name Supplementary Movie 6.

Description: PI3Kα-inactivated endothelial cells fail to stabilize nascent cell connections during anastomosis in zebrafish embryos. Time-lapse series (starting from 30 hpf) of the lateral view of a Tg(UAS:EGFP-UCHD) ubs18, Tg(kdrl:mCherry-CAAX) s916 embryo treated with 50 μM GDC-0326 from 27 hpf. Confocal stacks were acquired every 15 min. Endothelial cell membrane is visualized in red and actin cytoskeleton is visualized by F-actin binding domain of utrophin in green. We observe that upon PI3Kα inactivation two neighbouring tip cells establish an initial inefficient contact, retract and lead to the formation of a discontinuous DLAV compared to wild type sibling.

File Name: Supplementary Data 1

Description: Excel table with the analysed dataset of the 6,836 phosphopeptides obtained from the mass spectrometry analysis, with quantitative and statistical information. Related to Figure 4.
